# *Clostridium butyricum* isolated from giant panda can attenuate dextran sodium sulfate-induced colitis in mice

**DOI:** 10.3389/fmicb.2024.1361945

**Published:** 2024-04-05

**Authors:** Shuran Yu, Junjin Xie, Qiang Guo, Xia Yan, Yuxiang Wang, Tangjian Leng, Lin Li, Jielong Zhou, Wenping Zhang, Xiaoyan Su

**Affiliations:** ^1^College of Life Science, Southwest Forestry University, Kunming, China; ^2^Sichuan Key Laboratory of Conservation Biology for Endangered Wildlife, Chengdu Research Base of Giant Panda Breeding, Chengdu, China; ^3^College of Life Science and Biotechnology, Mianyang Normal University, Mianyang, China; ^4^College of Biodiversity Conservation, Southwest Forestry University, Kunming, China

**Keywords:** *Clostridium butyricum*, murine colitis, immune response, intestinal barrier, gut microbiota

## Abstract

**Objective:**

Probiotics are beneficial to the intestinal barrier, but few studies have investigated probiotics from giant pandas. This study aims to explore the preventive effects of giant panda-derived *Clostridium butyricum* on dextran sodium sulfate (DSS)-induced colitis in mice.

**Methods:**

*Clostridium butyricum* was administered to mice 14 days before administering DSS treatment to induce enteritis.

**Results:**

*Clostridium butyricum* B14 could more effectively prevent colitis in mice than *C. butyricum* B13. *C. butyricum* B14 protected the mouse colon by decreasing the histology index and serum interleukin-6 (IL-6) and tumor necrosis factor alpha (TNF-α) levels, which improved intestinal inflammation-related symptoms. In addition, the treatment led to the regulation of the expression of *Tifa, Igkv12-89,* and *Nr1d1*, which in turn inhibited immune pathways. The expression of *Muc4*, *Lama3, Cldn4, Cldn3*, *Ocln*, *Zo1, Zo2*, and *Snai* is related the intestinal mucosal barrier. 16S sequencing shows that the *C. butyricum* B14 significantly increased the abundance of certain intestinal probiotics. Overall, *C. butyricum* B14 exerted a preventive effect on colitis in mice by inhibiting immune responses, enhancing the intestinal barrier and increasing the abundance of probiotic species. Thus, *C. butyricum* B14 administration helps regulate the balance of the intestinal microecology. It can suppress immune pathways and enhance barrier-protective proteins.

## 1 Introduction

The giant panda (*Ailuropoda melanoleuca*) is a first-class national protected animal in China and a flagship species for global biodiversity conservation (Yang et al., 2022). However, it is also susceptible to various intestinal diseases, such as intestinal inflammation (Loeffler et al., 2006). Therefore, the treatment of intestinal inflammation in giant pandas warrants attention. When giant pandas are infected with pathogens, the integrity of the intestinal epithelium is disrupted with increases in permeability that cause antigenic substances to enter the intestinal mucosa and produce intestinal inflammation (Li J. et al., 2020; Zhou et al., 2021, 2023). In addition, undigested bamboo fibers in the gut of giant pandas may cause epithelial damage and parasitic infections (Williams et al., 2016; Wang et al., 2018; Qin et al., 2021), and during inflammation, these fibers may increase damage to the intestinal barrier and intestinal cell injury (Williams et al., 2016; Zhou et al., 2023). Inflammation can also accompany dysbiosis of the intestinal microbiota (Brandl and Schnabl, 2015).

Intestinal inflammation, such as colitis, is a common intestinal disease whose treatment largely depends on antibiotic administration. Overuse of antibiotics can cause imbalance in the intestinal microbiota, posing a potential threat to animal and human health. For example, antibiotic use can cause intestinal flora disorder in mice and result in increased mice susceptibility to some pathogenic strains intestinal colonization (Sekirov et al., 2008; Huang C. et al., 2022; Huang G. et al., 2022). Except for the impact on animals, the misuse of antibiotics may cause celiac disease, obesity, and attention deficit hyperactivity disorder in children (Aversa et al., 2021). Furthermore, overuse of antibiotics can enhance the prevalence of drug resistance genes, resulting in poorer treatment outcomes. Antibiotic-Resistant *Escherichia coli* and multidrug-resistant *Klebsiella pneumoniae* and *Raoultella* have been screened from the intestinal microbiota of giant pandas (Fan et al., 2022; Shahi et al., 2023). Therefore, it is particularly important to find a more effective way to treat intestinal diseases. Studies have shown that probiotics play an important role in intestinal diseases such as enteritis. Probiotics are considered to have beneficial effects on the host by regulating overall immune function, improving the balance of intestinal microbial nutrition, inhibiting the proliferation of harmful bacteria, reducing the colonization by pathogens, improving the function of the intestinal barrier, and maintaining the balance of the intestinal symbiotic microbial community (Mazziotta et al., 2023; Wolfe et al., 2023). Therefore, probiotics are a favorable option for treating conditions, such as intestinal inflammation.

The main metabolite of *C. butyricum* is butyric acid, and it has been found that butyrate can provide energy for the body through fatty acid oxidation, regulate body health, and play an important role in inhibiting intestinal inflammation and cancer (Sonia Archer et al., 1998; Segain et al., 2000; Tong et al., 2004; Zhang et al., 2021). Study found *C. butyricum* can inhibit pathogenic bacteria and exert preventive and therapeutic effects on gastrointestinal infections (Ariyoshi et al., 2022), including acquired intestinal infections, intestinal injuries, irritable bowel syndrome, inflammatory bowel disease, neurodegenerative and metabolic diseases, and colorectal cancer (Stoeva et al., 2021). In addition, some *C. butyricum* has been used in the treatment of intestinal inflammation-related cancers (Xiao et al., 2017; Liu et al., 2020). However, minimal knowledge of *C. butyricum*, especially regarding preventive and therapeutic effects on gastrointestinal infections, is available for giant pandas (Huang C. et al., 2022; Huang G. et al., 2022).

Herein, *C. butyricum* strains were isolated from giant panda feces to investigate their preventive effects on DSS-induced mouse colitis. The results indicated that *C. butyricum* B14 isolated from giant panda had a significant ability to control the increase in DSS-induced inflammation in a murine model. These findings can help identify favorable probiotics for the prevention of intestinal diseases of the giant panda and understand the mechanism by which *C. butyricum* B14 protects the health of the host.

## 2 Materials and methods

### 2.1 Preparation of the bacterial suspensions

Two stains, B13 and B14, of *C. butyricum* were isolated from giant panda feces (Mixed samples of 4 adult giant pandas (two males and two females) were enriched in M17 medium and then screened for two strains using a special medium for *Clostridium butyricum* (RCM) depending on the amount of butyric acid produced by following a novel pipeline of culturomics (unpublished data). A commercial strain of *C. butyricum* (denoted CB) was used to compare its results with those of B13 and B14. An inoculum loop of each *C. butyricum* stored at −80°C was streaked on reinforced Clostridium medium (RCM) agar and incubated in a Bactron Anaerobic Chamber at 37°C under anaerobic conditions (89.9% N_2_, 5.1% CO_2_, and 5% H_2_) for 48 h. A single colony of each strain was then anaerobically grown in RCM in the Bactron Anaerobic Chamber (89.9% N_2_, 5.1% CO_2_, and 5% H_2_) at 37°C for 24 h and subcultured three times. The concentration of each strain was adjusted to 1 × 10^9^ CFU/mL, which was used to gavage the mice.

### 2.2 Prevention of DSS-induced colitis by *Clostridium butyricum* interventions

Specific pathogen-free female C57BL/6 J mice (6–8 weeks old, purchased from Chengdu Dashuo Animal Technology Co., Ltd.) were acclimated for 7 days (23°C ± 2°C, 50% ± 10% relative humidity). All experimental procedures were approved by the Cheng du Research Base of Giant Panda Breeding Institutional Animal Care and Use Committee (SCXK 202015) and performed in accordance with internationally accepted guidelines and ethical principles.

**Figure 1:**
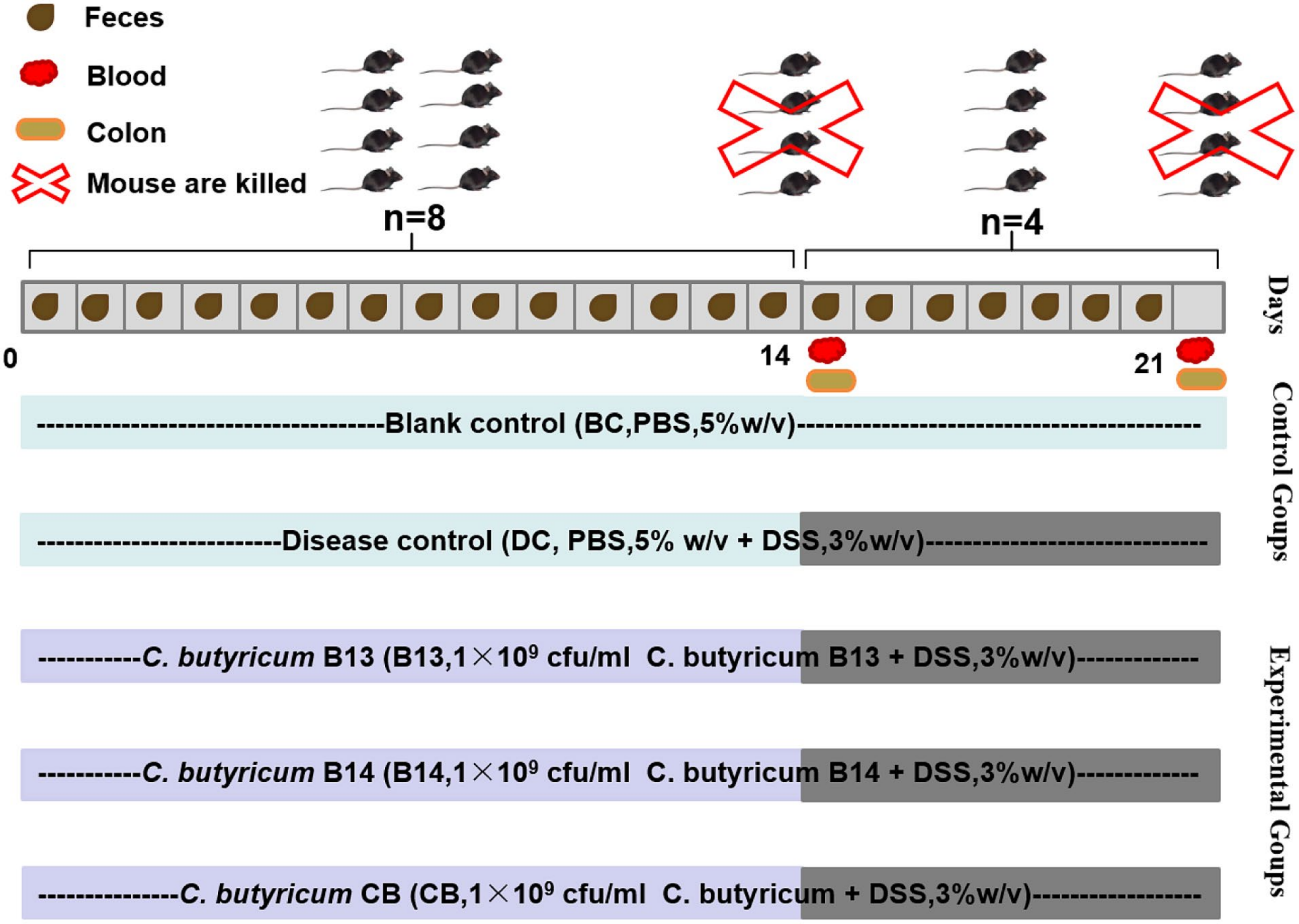
Establishment of dextran sodium sulfate (DSS)-induced ulcerative colitis in mice and experimental protocols for *C. butyricum* interventions. BC, Blank control group; DC, Disease control group; B13, *C. butyricum* B13 group; B14, *C. butyricum* B14 group; CB, *C. butyricum* CB group.

The preventive ability of *C. butyricum* was evaluated in DSS-induced ulcerative colitis in C57BL/6 J mice for 21 days (Figure 1). Mice were randomly divided into five groups: blank control (BC), disease control (DC), *C. butyricum* B13 (B13), *C. butyricum* B14 (B14), and *C. butyricum* CB (CB). Mice in BC and DC groups were orally administrated with PBS 200 μL/d and in groups B13, B14, and CB were orally administered with *C. butyricum* B13, B14, and CB (200 μL/d, 1 × 10^9^ cfu/mL) for 14 days, respectively (Figure 1). From day 14 to day 21, DSS (36000–5,000 Da, Yeasen Biotechnology, Shanghai, Co., Ltd) (3% w/v) was dissolved in drinking water to induce inflammation, except for BC. There were 8 mice in each group, and 4 mice were killed in each group before being fed with DSS (Figure 1).

Body weight was monitored throughout the study. Fresh feces were collected daily in sterile centrifuge tubes, snap-frozen, and stored at −80°C for 16S rRNA gene sequencing (Figure 1). DAI (Disease Activity Index) score is calculated based on this formula: DAI score = (body weight loss score + fecal status score + rectal bleeding score) / 3 (Supplementary Table S1). Mice were euthanized by spinal cord dislocation, and colon tissue and blood collected (Figure 1).

### 2.3 Histopathological evaluation of the colon tissue

A segment of colon tissue located 1 cm from the mouse anus was rinsed with PBS. The colon portion was fixed using 4% paraformaldehyde for 48 h at 4°C. Fixed segments were paraffin-embedded, cut into 5-μm sections, and stained with hematoxylin and eosin (Morin et al., 2016). Sections were scanned using a digital slide scanner for image acquisition and histopathological analysis, following the criteria listed in Supplementary Table S2.

### 2.4 Enzyme-linked immunosorbent assay (ELISA) of serum

Inflammation was assessed by measuring IL-1β, IL-6, and TNF-α levels in the serum using commercially available ELISA kits (Shanghai Enzyme-linked Biotechnology Co., Ltd).

### 2.5 Gene expression analysis

RNA was extracted from colon tissue using the SPINeasy RNA Kit for Tissue (MP Biomedicals) and was converted to cDNA using Exon Script RT Super Mix with dsDNase kit (Catalog no: A502-02). cDNA was used for transcriptome sequencing and to evaluate the expression of *Cldn*3, *Ocln*, *Zo*1, *Zo*2.

cDNA prepared from DC, B14, and CB colon tissues were selected for transcriptome sequencing. Paired-end libraries were prepared using an ABclonal mRNA-seq Lib Prep Kit (ABclonal, China), and library quality was assessed using an Agilent Bioanalyzer 4,150 system. Finally, the library preparations were sequenced on an Illumina MGISEQ-T7 and 150-bp paired-end reads were generated. Low-quality reads and any adapter sequences were removed as previously described (Zhao et al., 2013). High-quality reads were mapped to the reference genome^1^ in orientation mode using HISAT2 software (Kim et al., 2015). The final efficiency of RNA-seq read alignments varied from 96.91 to 98.40% (Supplementary Table S3). Differentially expressed genes (DEGs; | log_2_FC | > 1 and *p*_adj_ < 0.05) were analyzed using DESeq2 (Love et al., 2014). Gene ontology (GO) and pathway enrichment analysis of DEGs was performed using the “enricher” function in the “clusterProfler” package in R (Yu, 2018).

Fast SYBR Green qPCR Master Mix UDG (Catalog no: A402-01) was used for RT-PCR. Negative controls containing water as a template and the housekeeping gene *Gapdh* as an internal control were included in all RT-qPCR runs. The 2^−ΔΔCt^ method was used to calculate the relative gene expression based on *Gapdh* expression. The gene primer sequences of *Cldn3*, *Ocln*, *Zo*1, and *Zo2* were found in PrimerBank and are shown in Supplementary Table S4. According to the instructions, the cycling conditions included: 1 cycle of 50°C for 120 s; 1 cycle of 94°C for 600 s; 1 cycle of 95°C for 180 s; 40 cycles of 95°C for 5 s and 60°C for 30 s.

### 2.6 16S rRNA gene sequencing and analysis

Total DNA from the feces samples was extracted using a Magnetic Soil and Stool DNA Kit (TIANGEN). DNA concentration and purity were monitored on 1% agarose gels. V3 + V4 regions of 16S rRNA genes were PCR amplified using a pair of universal primers (Forward: CCTAYGGGRBGCASCAG, Reverse: GGACTACNNGGGTATCTAAT) tagged with 6-bp barcodes (Zhai et al., 2019). PCR conditions were 94°C for 4 min, followed by 30 cycles of 94°C for 30 s, 54°C for 30 s and 72°C for 30 s and then 72°C for 5 min. Single amplification was performed in 25-μL reactions with 50 ng of template DNA with the Phusion^®^High-Fidelity PCR Master Mix (New England Biolabs). Sequencing libraries were generated using the TruSeq^®^ DNA PCR-Free Sample Preparation Kit (Illumina). Paired-end reads were obtained using the Illumina MiSeq PE-250 platform according to the standard protocols of Shanghai Applied Protein Technology Co., Ltd. Microbial raw sequences were analyzed using the QIIME2 pipeline (version 2021.2) (Bolyen et al., 2019). Paired-end reads were merged using FLASH (version 1.2.7) (Magoč and Salzberg, 2011). The DADA2 plugin was used to denoise and quality filter reads, and a feature table of amplicon sequence variants (ASVs) was prepared for subsequent analysis. After selecting the representative sequences of each ASV using QIIME 2 software, all representative sequences were aligned to the database for annotation against the SILVA reference database (version 138) (Pruesse et al., 2007) in QIIME2. Based on the ASV cluster analysis results and annotation information, the α diversity index, β diversity index, and species abundance at each taxonomic level were analyzed using Microbiome Analyst (Dhariwal et al., 2017).^2^

### 2.7 Statistical analysis

Differences between groups were analyzed via one-way analysis of variance using the IBM SPSS Statistics 27 software with least-squares deconvolution. Data are expressed as mean ± SEM. The LEfSe of the Huttenhower Galaxy Server^3^ was also used to compare the differences in gut microbiota among groups (*p* < 0.05; LDA score > 2.0).

## 3 Results

### 3.1 *Clostridium butyricum* interventions decreased harmful changes in the colon

At day 14, i.e., when no DSS were administered into mice, supplementation with *C. butyricum* had no significant effect on hematoxylin and eosin staining of colonic tissues (Supplementary Figure S1).

**Figure 2:**
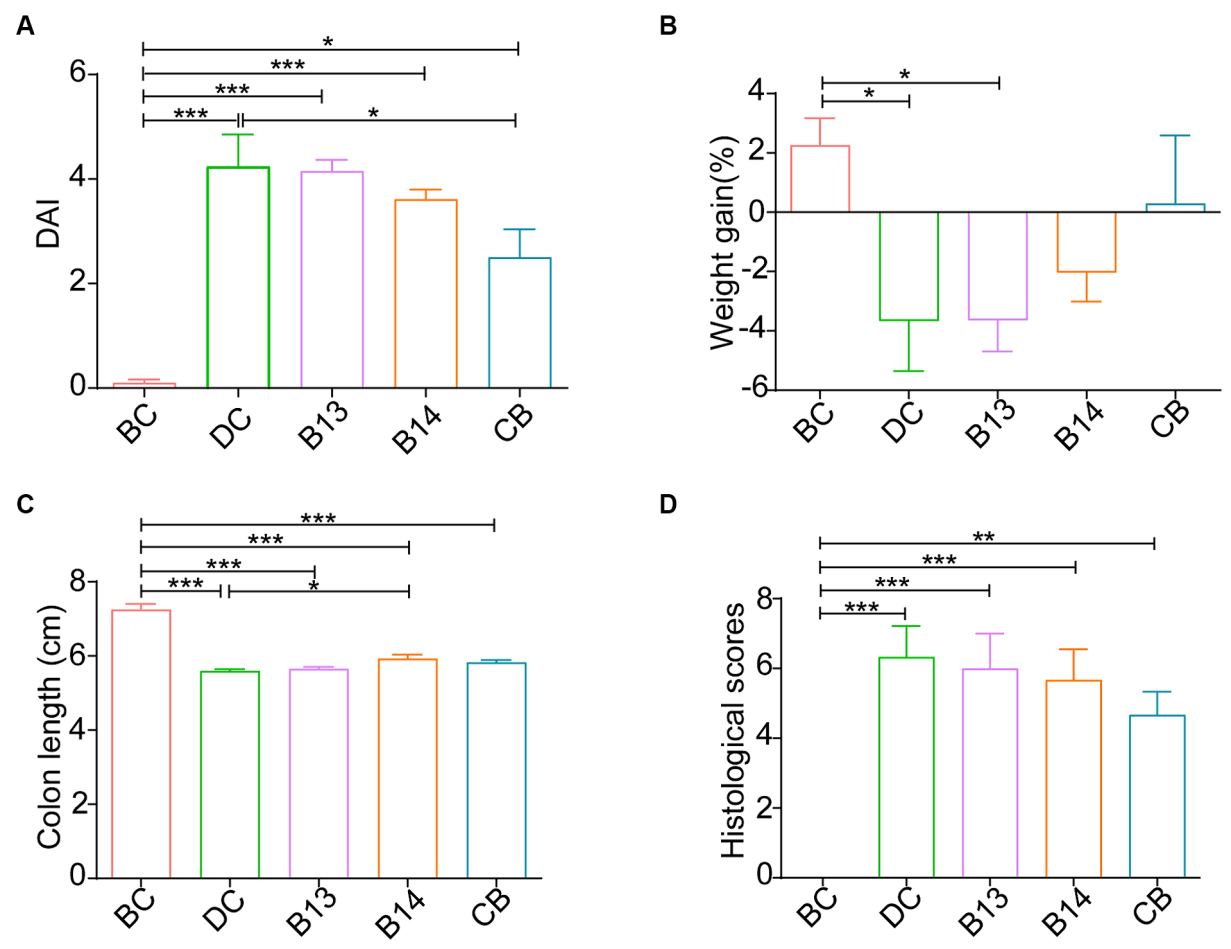
*C. butyricum* interventions protected mice against DSS-induced harmful changes in the colon on day 21. Disease activity index (DAI) **(A)**, Weight gain **(B)**, colon length **(C)** and mouse colitis histology index **(D)**. BC, Blank control group; DC, Disease control group; B13, *C. butyricum* B13 group; B14, *C. butyricum* B14 group; CB, *C. butyricum* CB group. Data are expressed as mean ± SEM (*p < 0.05, **p < 0.01, ***p < 0.001).

**Figure 3:**
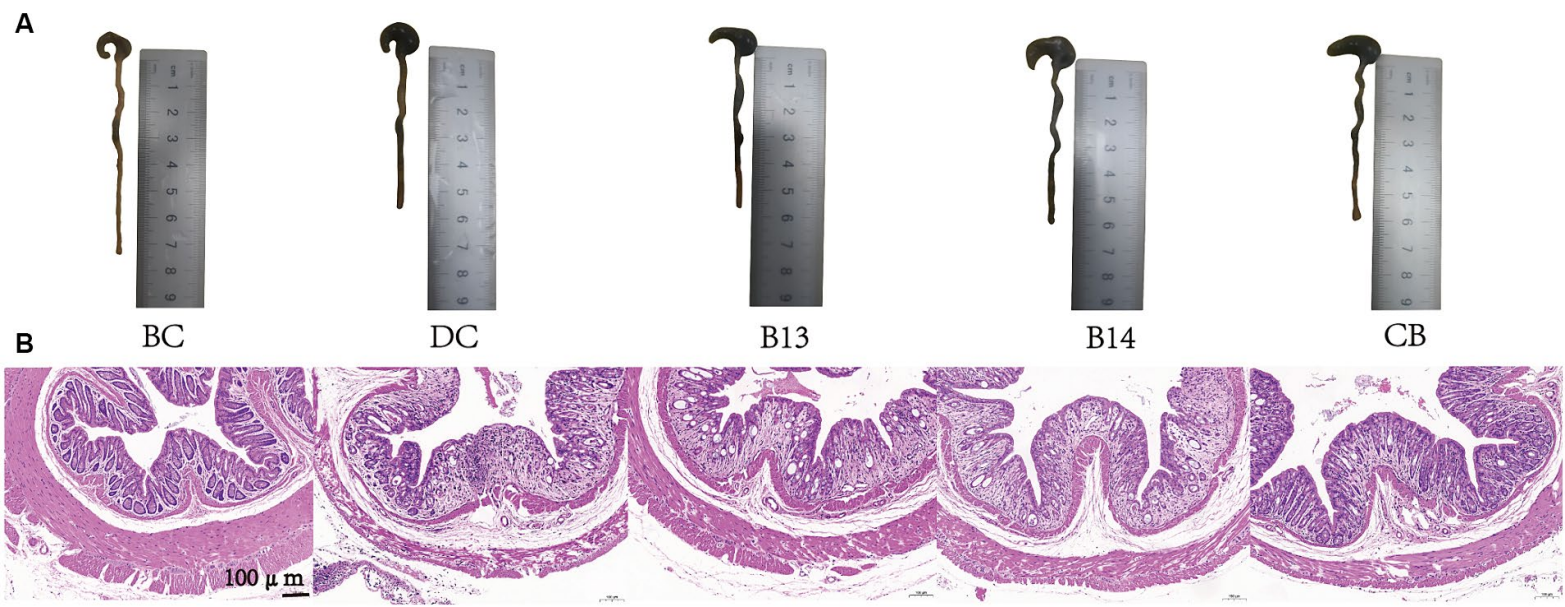
*C. butyricum* interventions protected mice against DSS-induced harmful changes in the colon on day 21. Colon length images **(A)** and hematoxylin and eosin staining of colonic tissues **(B)**. BC, Blank control group; DC, Disease control group; B13, *C. butyricum* B13 group; B14, *C. butyricum* B14 group; CB, *C. butyricum* CB group.

On day 21, after DSS had been administered for 7 days, significant damage to mouse colons was observed. The BC group (without DSS) had the lowest DAI (*p* < 0.05), the longest colon length (*p* < 0.001), and the lowest histological scores (*p* < 0.01) among the five groups (Figures 2, 3). In the absence of *C. butyricum* interventions, the DC group had the highest DAI score, lowest weight gain, shortest colon length, and highest histological scores among the five groups (Figures 2, 3). Among the three groups B13, B14, and CB with *C. butyricum* interventions, the CB group had a significantly lower DAI score and higher weight gain than that of the DC group (*p* < 0.05) but with no significant difference between B13 and B14 (Figures 2A,B). The B14 and CB groups had a significantly longer colon length than the DC group but did not significantly differ between each other (*p* > 0.05) (Figure 2C); the CB group had the lowest histological scores but was not significantly different from other groups (Figure 2D). Thus, *C. butyricum* could enhance the integrity of colonic mucosa and decrease tissue damage in DSS-induced mice; furthermore, administration of *C. butyricum* B14 and CB produced more protective effects than that of *C. butyricum* B13.

### 3.2 *Clostridium butyricum* interventions decreased inflammation in the serum

**Figure 4:**
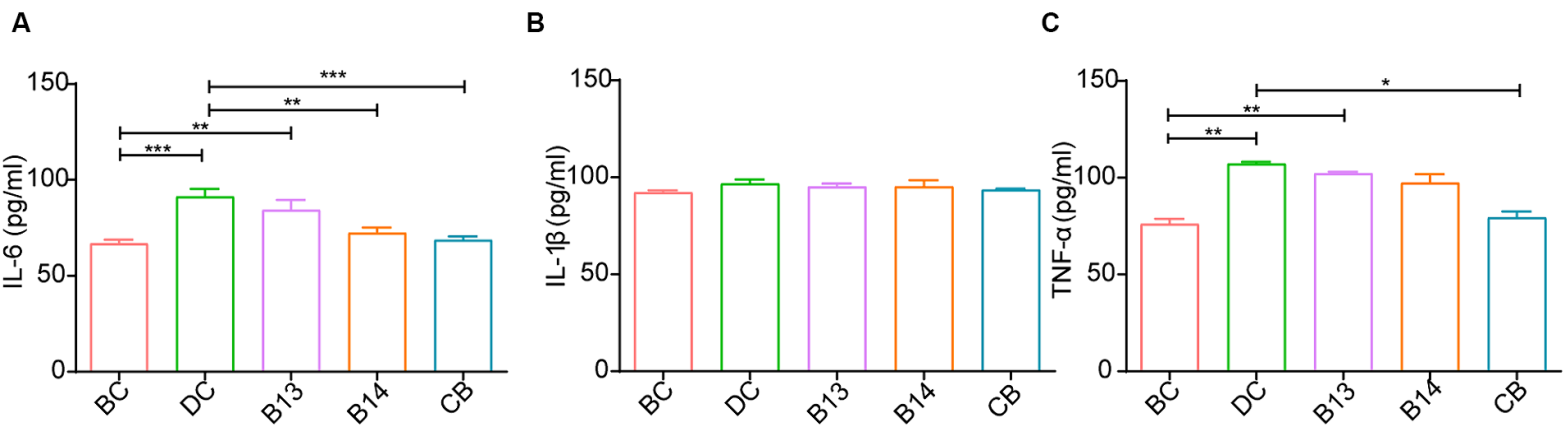
Effect of *C. butyricum* on inflammatory factor levels in serum of DSS-induced mice colitis. IL-6 **(A)**, IL-1β **(B)**, and TNF-α **(C)**. BC, Blank control group; DC, Disease control group; B13, *C. butyricum* B13 group; B14, *C. butyricum* B14 group; CB, *C. butyricum* CB group. Data are expressed as mean ± SEM (*p < 0.05, **p < 0.01, ***p < 0.001).

The serum levels of IL-6 and TNF-α was significantly higher following DSS treatment in the DC group than that in the BC group (*p* < 0.05); no significant difference was present between the DC and BC groups for IL-1β without the protection of *C. butyricum* (Figure 4). The *C. butyricum* intervention significantly decreased the serum levels of IL-6 in B14 and CB and TNF-α in the CB group compared with those in the DC group (*p* < 0.01 and *p* < 0.05, respectively), but serum IL-6, IL-1β, and TNF-α levels in the B13 group were not significantly decreased (Figure 4). The differences in IL-6, IL-1β, and TNF-α lacked significant difference among B13, B14, and BC groups, although the B13 group had a relative higher value than the other two groups (Figure 4). The levels of serum IL-6 and TNF-α in B13 were significantly higher than those in the BC group (*p* < 0.01), but the differences in the levels of IL-6 and TNF-α were not significantly different between BC and B14 or CB groups (Figure 4). The difference in the level IL-1β did not significantly differ among the five groups (Figure 4B). Thus, *C. butyricum* B14 and CB could significantly decrease the inflammation in DSS-induced colitis in mice.

### 3.3 *Clostridium butyricum* can influence gene expression in the colon tissue of mice with DSS-induced colitis

The above results show that *C. butyricum* B14 and CB had favorable protective effects against DSS-induced colitis in mice. Therefore, we used transcriptomic profiling to analyze the impact of *C. butyricum* B14 and CB on colitis development. RNA extracted from colon tissue from adult male mouse was sequenced using DNBSEQ-T7. Sequencing generated 66.12G clean data for mouse samples. The sequences of each sample were the aligned to the genome sequence of the respective species. RNA-seq read alignment efficiency varied from 96.91 to 98.4% (Supplementary Table S5).

**Figure 5:**
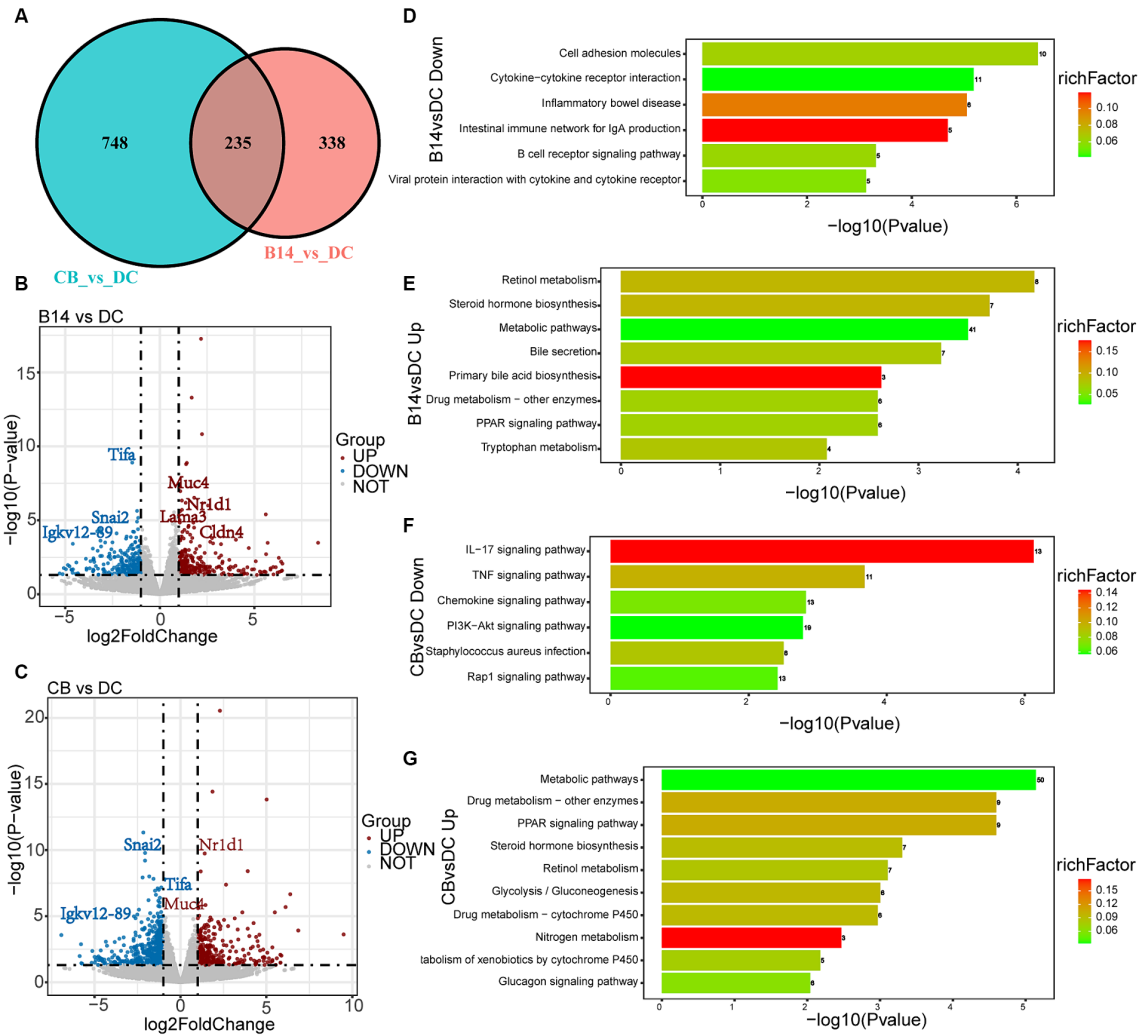
Transcriptome analysis of DSS-induced mice exposed to *C. butyricum*. Venn diagram of the DEGs between DC, B14, and CB groups **(A)**. Volcano plot shows the DEGs in B14 vs. DC **(B)** and CB vs. DC **(C)**. Related downregulated pathways for KEGG enrichment based on DEGs in B14 vs. DC **(D)** and CB vs. DC **(F)**. Related upregulated pathways for KEGG enrichment based on DEGs in B14 vs. DC **(E)** and CB vs. DC **(G)**. BC, Blank control group; DC, Disease control group; B13, *C. butyricum* B13 group; B14, *C. butyricum* B14 group; CB, *C. butyricum* CB group.

A total of 573 DEGs were identified in B14 vs. DC, of which 292 were upregulated and 281 were downregulated (Figure 5B). A total of 983 DEGs were identified in CB vs. DC, of which 352 were upregulated and 631 were downregulated (Figure 5C). Among these DEGs, 235 DEGs overlapped between the two groups (Figure 5A). The top 30 GO terms of these DEGs were related to the immune system, inflammatory, and metabolic activity (Supplementary Figure S2).

Kyoto Encyclopedia of Genes and Genomes (KEGG) pathway enrichment analysis was performed, and the top 20 KEGG pathways of DC vs. B14 showed that the downregulated pathways were related to immune and inflammatory processes, such as cell adhesion molecules, inflammatory bowel disease, and B cell receptor signaling pathway and the upregulated pathways were related to metabolic activity pathways, such as retinol metabolism, steroid hormone biosynthesis, metabolic pathways, and PPAR signaling pathway (Figures 5D,E). Similar to DC vs. B14, downregulated pathways in DC vs. CB were enriched in immune and inflammatory pathways, including the IL-17 signaling pathway, TNF signaling pathway, and chemokine signaling pathway, and upregulated pathways were enriched in metabolic activity pathways, including metabolic pathways, Drug metabolism-other enzymes, and PPAR signaling pathway (Figures 5F,G). In summary, *C. butyricum* B14 and CB could downregulate inflammation-related pathways and upregulate metabolism-related pathways to protect the host and decrease the inflammation level induced by DSS in mice models.

**Figure 6:**
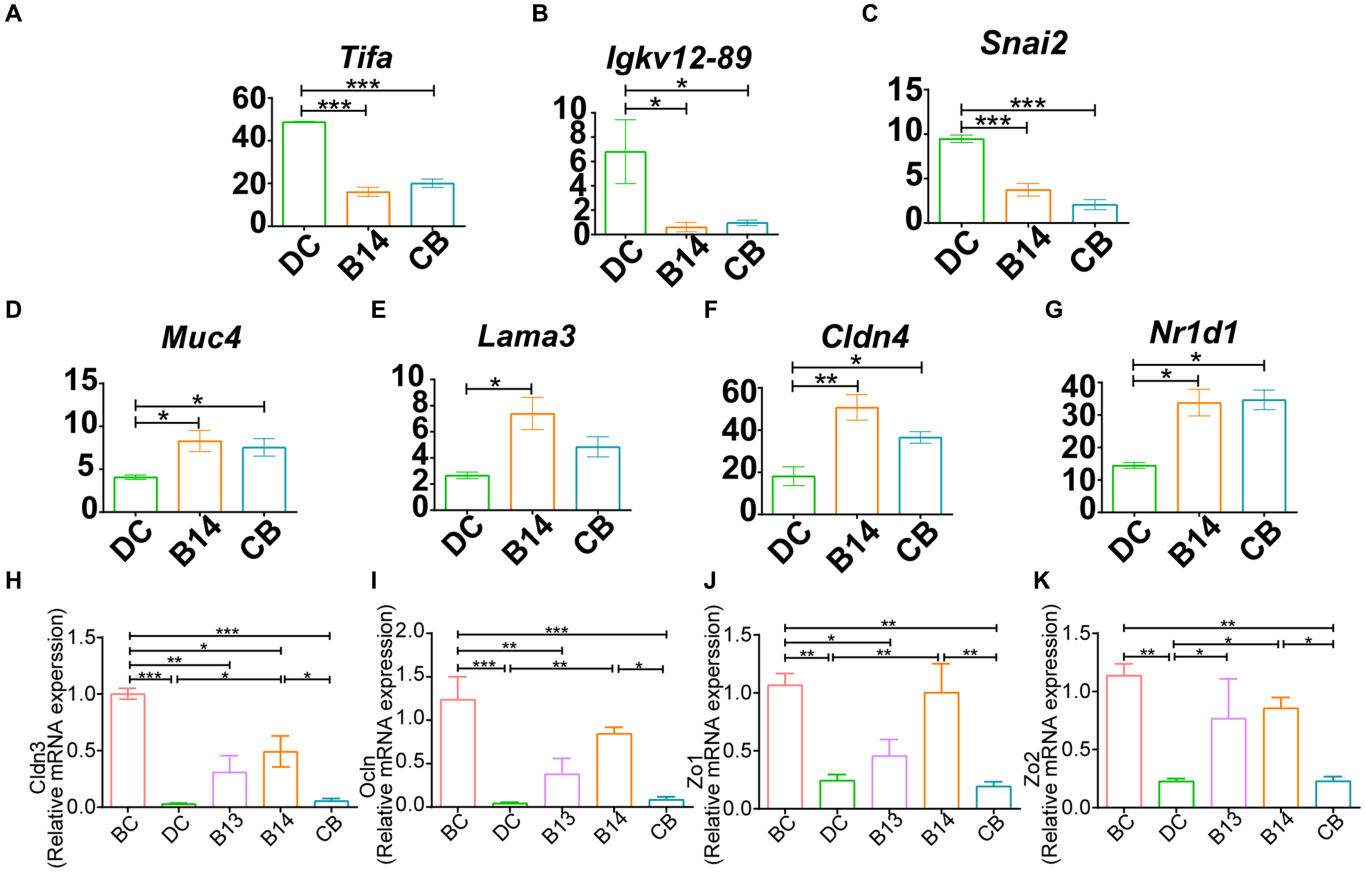
B14 and CB share significantly different related DEGs. Downregulated DEGs, including *Tifa* **(A)**, *Igkv12-89* **(B)** and *Snai2* **(C)** and upregulated DEGs, including *Muc4* **(D)**, *Lama3* **(E)**, *Cldn4* **(F)**, and *Nr1d1* **(G)**. qPCR analysis of the expression of tight junction genes in colon tissue. *Cldn3* **(H)**, *Ocln* **(I)**, *Zo1* **(J)**, and *Zo2* **(K)**. BC, Blank control group; DC, Disease control group; B13, *C. butyricum* B13 group; B14, *C. butyricum* B14 group; CB, *C. butyricum* CB group. Data are expressed as mean ± SEM (*p < 0.05, **p < 0.01, ***p < 0.001).

Among these DEGs, *Tifa* and *Igkv12-89*, which are involved in inflammatory and immune-related signaling pathways, and S*nai2*, which is related to the intestinal barrier, had a significantly lower expression in the B14 and CB groups (*p* < 0.05) (Figures 6A–C), and upregulated DEGs, including *Muc4*, *Lama3*, and *Cldn4*, are related to *Nr1d1,* which is associated with the intestinal barrier and is involved in immune-related signaling pathways (*p* < 0.05) (Figures 6D–G). These seven genes should be the key genes contributing to the decrease in inflammation in DSS-induced mice for *C. butyricum* B14 and CB.

The DEGs excluded the intestinal barrier-associated genes *Cldn3*, *Ocln*, *Zo*1, or *Zo*2; therefore, qPCR was subsequently used to detect the expression levels of these four genes to evaluate the mucosal barrier function in the intestine (Figures 6H–K). Expression levels of these genes in the DC group treated with DSS but not with *C. butyricum* intervention was significantly decreased compared with those in the BC group (*p* < 0.01) (Figures 6H–K). Compared with that in the DC group, intervention with *C. butyricum* B14 did not significantly increase the expression of these genes, whereas they were significantly increased with *C. butyricum* B14 treatment (*p* < 0.05); *C. butyricum* B13 only significantly increased the expression of *Zo2* (*p* < 0.05) (Figures 6H–K). Thus, *C. butyricum* B14 had the greatest potential to decrease the damage induced by DSS in the colon.

### 3.4 *Clostridium butyricum* can modulate the composition of the gut microbiota

16S rRNA amplicon sequencing was used to analyze the microbiota in the 40 fecal samples on day 14 and 21^t^ in the five groups (Supplementary Table S6). A total of 4,875,721 high-quality 16S rRNA gene sequences were obtained by quality filtering and paired-end sequence assembly, averaging 121,893 per sample, and ranging from 97,622 to 139,137 (Supplementary Table S6). These 4,875,721 reads were classified into 7,989 ASVs, belonging to 35 phyla (Firmicutes, Bacteroidetes, etc.) and 384 genera.

**Figure 7:**
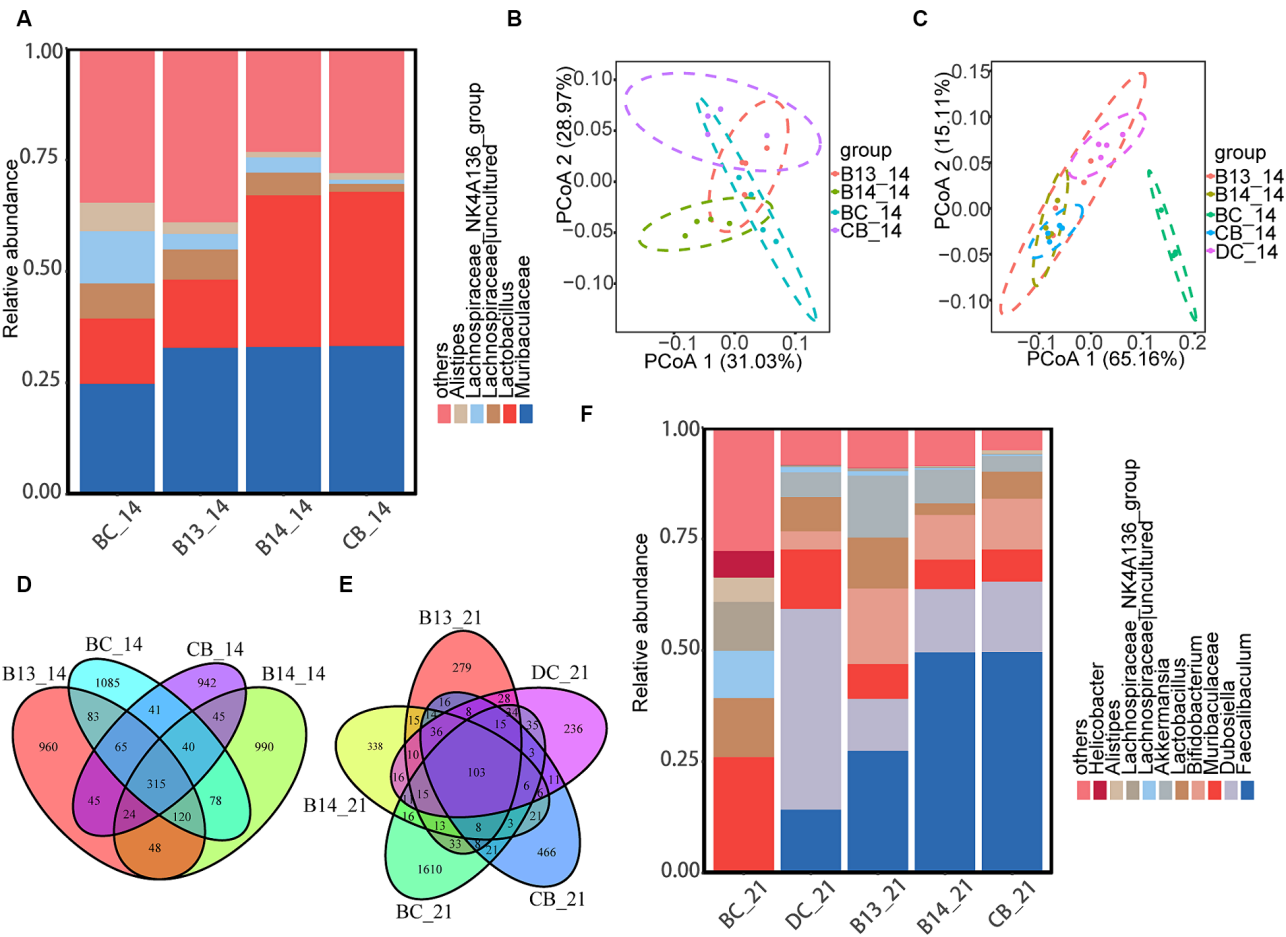
*C. butyricum* supplementation modulated the composition of colonic microbiota. At the genus level, the heatmap of the abundant colonic microbiota on day 14 **(A)** and five groups on day 21 **(F)**. PCoA plot of the gut microbiota based on weighted UniFrac distance among the four groups on day 14 **(B)** and five groups on day 21 **(C)**. Venn diagram showing the overlap of the ASVs identified in the intestinal microbiota among the four groups on the day 14 **(D)** and five groups on day 21 **(E)**. BC, Blank control group; DC, Disease control group; B13, *C. butyricum* B13 group; B14, *C. butyricum* B14 group; CB, *C. butyricum* CB group.

At the phylum level, the predominant bacterial communities were Firmicutes and Bacteroidetes in mice (Supplementary Figures S3A,C). At the genus level, before DSS administration, treatment with *C. butyricum* decreased the relative abundance of *Alistips*, especially in the B14 and CB groups, where the relative abundance of *Alistips* in B14_14 and CB_14 was lower than that in BC_14 (*p* < 0.05) (Figure 7A).

Following DSS administration, the relative abundance of *Bifidobacterium* and *Faecalibaculum* was significantly higher in B14_21 and CB_21 than in DC_21 (*p* < 0.05) (Figure 7F). In addition, the relative abundance of *Dubosiella* in the three experimental groups with *C. butyricum* intervention was significantly lower than that in DC_21 (*p* < 0.05) (Figure 7F). Moreover, the relative abundance of *Actinobacteriota* was significantly increased in B14_21 and CB_21 compared with that in DC_21 (*p* < 0.05) (Supplementary Figure S3).

The Venn diagram (Figure 7D) shows that 315 ASVs were shared between mice on day 14 before exposure with DSS and only 103 common ASVs were shared between mice after DSS administration on day 21 (Figure 7E). The α-diversity results showed no significant difference in the ACE, Chao1, Shannon, or Simpson indices among the five groups on day 14 (*p* > 0.05) (Supplementary Figures S3A,C). In contrast to BC_14, the gut microbiota richness did not significantly differ after the administration of *C. butyricum* (Supplementary Figure S3B). Compared with BC_21, there was a significantly decreased gut microbiota richness in the other four groups after DSS administration (*p* < 0.05) (Supplementary Figure S3D). Compared with DC, only the Simpson index was significantly higher in B13 (*p* < 0.05), and no differences were observed in the other three indexes (*p* > 0.05) (Supplementary Figure S3D). In addition, no differences were observed in ACE, Chao1, Shannon, and Simpson indices between B14 and CB (*p* > 0.05) (Supplementary Figure S3D).

The principal coordinate analysis (PCoA) based on weighted UniFrac metrics showed that the B14 and CB groups formed two distinct clusters and that the BC and B13 samples had a larger overlap than samples from the B14 and CB groups on day 14 (Figure 7B); the gut microbiota in BC_21 was significantly different from the other four groups while CB_21 and B14_21 clustered together but were divided with DC_21 (Figure 7C).

LEfSe showed that the abundance of class *Clostridia* and genus *Akkermansia* in B14_14 dramatically increased compared with that in BC_14 (Supplementary Figures S4A,G). The CB_14 sample had an increase in the relative abundance of *Bacilli* at the class level and *Bifidobacterium* and *Faecalibaculum* at the genus level compared with that in BC_14 (Supplementary Figures S4B,H). Compared with that in the BC_21, sample the relative abundance of Erysipelotrichaceae increased at the family level in DC_21 (Supplementary Figures S4C,I). The abundance of *Dubosiella* significantly increased at the genus level in DC_21 (Supplementary Figures S4C,J), and the abundance of *Faecalibaculum* and *Bifidobacterium* at the genus level and Bifidobacteriaceae at the phylum level significantly increased in B14_21 and CB_21 (Supplementary Figures S4D,E,I,F).

## 4 Discussion

Enteritis is an intestinal disease that affects the health and metabolism of the host, and its symptoms include disruption of the intestinal immune system and intestinal barrier (Salim and Söderholm, 2011; Yu and Rodriguez, 2017). Currently, immunosuppressants, salicylic acid preparations, and glucocorticoids are used to regulate immunity and inhibit inflammation to alleviate symptoms. However, disadvantages of this treatment include high recurrence rates and low remission rates (Chen et al., 2022). Previous studies showed that probiotic intervention can effectively regulate intestinal inflammation and reduce the damage (Niu et al., 2023; Sharma et al., 2023). Therefore, probiotics have become an alternative for preventing and treating several diseases, such as enteritis. In addition, the DSS-induced mouse enteritis model is easy to operate and has been applied to many probiotics in evaluating the effects on colitis such as *Lactobacillus plantarum*, Bifidobacterium and so on (Niu et al., 2023; Wang et al., 2023). Consequently, a DSS-induced mouse enteritis model was used to study the effect of *C. butyricum* isolated from giant panda feces. Based on this mouse model study, our findings show that pretreatment with *C. butyricum* B14 and CB can increase the colon length of colitis mice, improve colon histological scores, and decrease the rate of weight loss caused by enteritis and the level of inflammation in serum. The underlying mechanism may be related to the downregulation of immune-related pathways and enhancement of intestinal barrier protection. The results of this study are similar to those of previous studies on the anti-inflammatory effects of probiotics (Wickramasinghe et al., 2015; Sharma et al., 2023).

### 4.1 *Clostridium butyricum* can downregulate the expression of inflammation-related pathway genes in the colon to decrease inflammation

**Figure 8:**
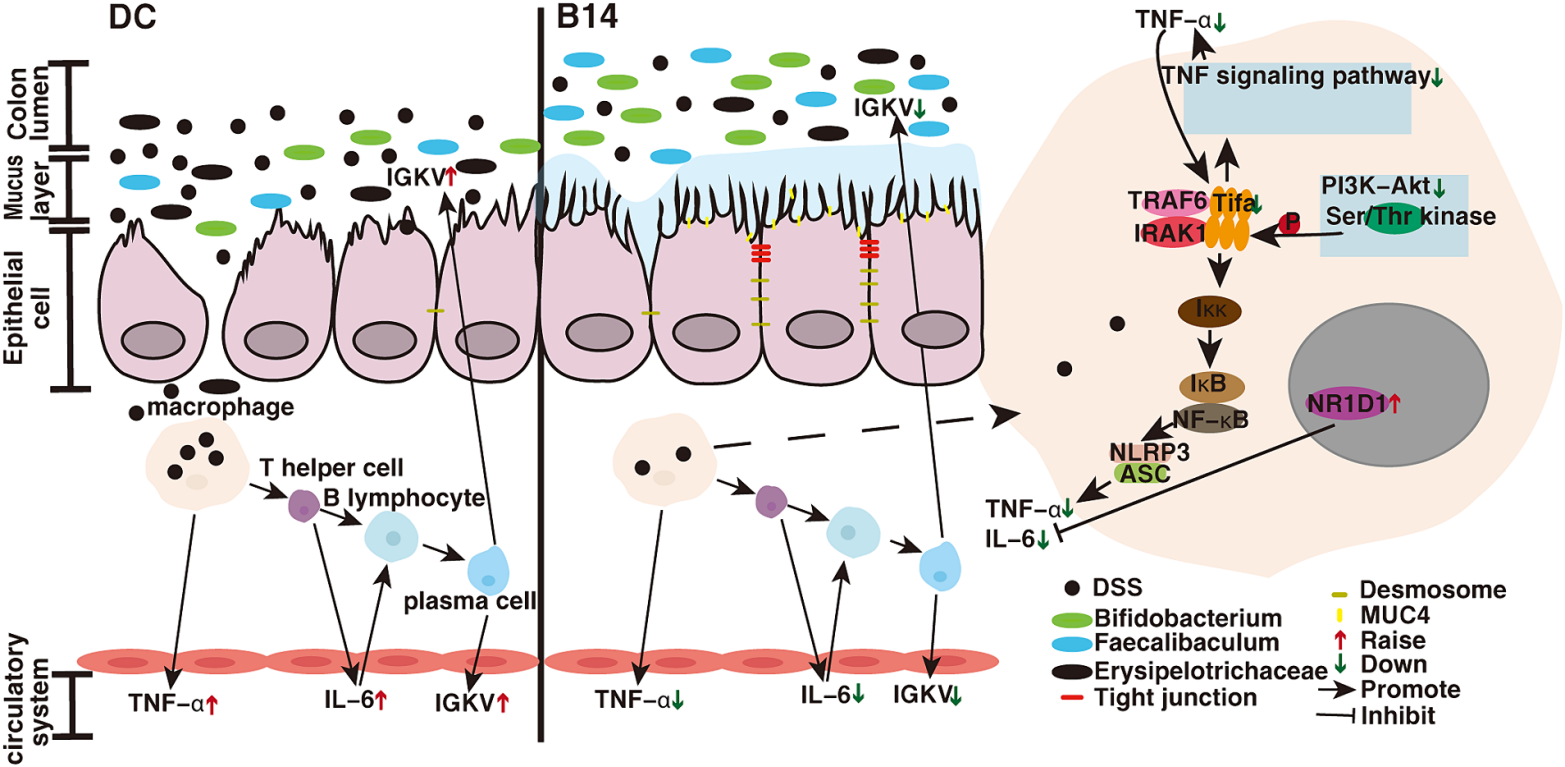
*C. butyricum* mechanistic diagram for the prevention of enteritis. DC, Disease control group; B14, *C. butyricum* B14 group.

Inflammation can activate the immune response and prompt sustained activity of the adaptive immune system (Hespel and Moser, 2012). In this study, we found that the levels of inflammatory factors were downregulated following treatment, indicating that the immune response may be suppressed. Therefore, we analyzed the DEGs in the DC, B14, and CB groups and identified several genes related to immune response, including *Tifa*, *Igkv12-89*, and *Nr1d1*. TIFA (T2BP) is an inflammatory signaling adaptor that includes an FHA domain (Shen et al., 2015). TIFAs are important proteins that link the TNF signaling and NF-κB pathways that are a part of the proinflammatory stress response (Kanamori et al., 2002); moreover, they are involved in innate immunity induced by pathogen-associated molecular patterns (Li Y. et al., 2023). TNF-α–mediated signaling is attenuated when endogenous *Tifa* is knocked out. However, TIFA dimers bind with each other through intermolecular FHA-pT9 upon stimulation with inflammatory cytokines, such as TNF-α, resulting in TRAF6 oligomerization and subsequent NF-κB activation (Huang et al., 2023) (Figure 8). The NF-κB pathway is a common immune response pathway (Aggarwal, 2006). We found that *Tifa* expression was significantly downregulated after the administration of *C. butyricum*, demonstrating that inflammatory pathways were indeed inhibited. The KEGG enrichment results also supported this conclusion. Another related gene, *Igkv12-89*, is downstream of immunomodulation (Brüggemann et al., 2019). *Igkv12-89* can be transcribed into IGKV12-89, which belongs to the immunoglobulin Kappa variable cluster (IGKV). During humoral immunity (induced by DSS or invasion of pathogenic bacteria), lymphocytes (e.g., macrophages) recognize and bind antigens, contact B cells to activate helper T lymphocytes, secrete interleukins and other cytokines, and together with T lymphocytes activate B cells to produce effector B cells that secrete IGKV (Catera et al., 2017) (Figure 8). Our results suggest that the downregulation of the signaling pathway upstream of TIFA may affect the downregulation of downstream *Igkv12-89* expression. We found that the gene expression of a negatively regulated immune system is upregulated-*Nr1d1* in our results. NR1D1 can negatively regulate the proinflammatory cytokine IL-6 in macrophages (Goswamy, 2022) (Figure 8), which is consistent with the reduced levels of IL-6 in our results. Zhao et al. (2019) show that *C. butyricum* can also reduce the level of the inflammatory factor IL-6. In addition, NR1D1 is a circadian clock component that integrates circadian rhythm and metabolism and plays a role in promoting metabolism (Everett and Lazar, 2014). According to KEGG analysis, most upregulated pathways are enriched in metabolism-related pathways, and we suspect that this may be related to the upregulation of *Nr1d1*. The effect of the above changes in these DEGs is consistent with the downregulation of the immune pathways shown in KEGG results. Studies have found that *C. butyricum* can inhibit the activation of pathways such as NF-κB and PI3K-Akt, thereby reducing the inflammatory response and protecting the intestine (Wang et al., 2022). Xie et al. (2020) research shows that after giving *C. butyricum* to mice with enteritis, its inflammation-related pathways are also significantly down-regulated, but mainly about IL-17 inflammatory factor-related pathways. Therefore, *Tifa* and *Nr1d1* can be considered as therapeutic targets for inflammatory/immune diseases. Thus, *C. butyricum* B14 and CB treatment reduced the expression of proinflammatory factors. When the expression of *Tifa* is downregulated, the TNF-α–mediated signaling is attenuated, inhibiting the expression of inflammatory cytokines (Huang et al., 2023). The downregulation of IGKV expression also indicates that *C. butyricum* downregulates humoral immunity. Therefore, the downregulation of *Tifa* and *lgkv12-89* and the upregulation of *Nr1d1* all reflected a reduction in the immune response.

### 4.2 *Clostridium butyricum* upregulates the expression of intestinal barrier-related pathway genes in the colon to decrease inflammation

TNF-α and IL-6 can exacerbate intestinal inflammation by increasing colonic mucosal permeability, disrupting the intestinal tight junction protein barrier, and increasing intestinal penetration of tubular antigens, in addition to being involved in regulating inflammatory responses (Al-Sadi et al., 2009; Farré et al., 2020). Basement membrane laminin, macrophage transmigration, and associated loss of the intestinal tight junction barrier are key factors in the induction of enteritis (Nighot et al., 2021). Our transcriptome results show that *Muc4*, *Lama3*, and *Cldn4*, which are related to the intestinal barrier, were significantly upregulated and that the B14 strain had a stronger effect than the B13 strain. The normal intestinal surface is covered with a layer of mucus that comprises secretory mucin, which forms an external loose layer and an internal dense layer, and membrane-bound mucin, which forms a glycocalyx covering and protects the surface epithelial cells (McGuckin et al., 2011). These glycoproteins play an important role in protecting epithelial cells and are associated with epithelial renewal and differentiation. When intestinal inflammation occurs, the intestinal mucin barrier is disrupted, which aggravates intestinal damage. Mucin-4 (Muc4) is a large transmembrane glycoprotein composed of a heavily O-glycosylated extracellular α subunit and membrane-anchored *β* subunit, which usually acts to lubricate and protect the apical epithelial surface (Das et al., 2015; Rowson-Hodel et al., 2017). Mucin molecules may also promote mucosal repair and healing (Longman et al., 2000). Disruption of the basement membrane (BM) barrier function leads to intestinal inflammatory diseases. Laminin332 is an important constituent protein of the BM (Tayem et al., 2021). The protein encoded by *lama3* is an α3 chain of laminin332 and plays a vital role in nucleation during hemidesmosome (HD) assembly and in maintaining the structural integrity of the HD (Schneider et al., 2007; Li Y. et al., 2020). When *lama3* is mutated or missing, complete loss of laminin332 can co-occur in the BM and affect HD formation, which may eventually cause borderline epidermolysis bullosa and an inflammatory phenotype (Herrmann et al., 2021). The processed peptide laminin, which is a component of the extracellular matrix, protects epithelial tissue from pathogen attack and is part of the innate defense mechanism (Senyürek et al., 2010). Laminin α3 expression and processing are enhanced after infection and in chronic wounds, resulting in faster wound healing (Senyürek et al., 2014). Laminin can impact the microenvironmental response to inflammation in the intestine and likely participate in the regeneration process. In transgenic mice overexpressing laminin, the inflammatory response to DSS was attenuated, further suggesting that it had a protective effect against enteritis (Ljubimov et al., 2014). In our study, the expression of *Lama3* was visibly elevated after the addition of *C. butyricum*, and *C. butyricum* B14 promoted the healing of damaged parts of enteritis. *Lama3* has also been shown to be expressed in Crohn’s disease (Bouatrouss et al., 2000). Moreover, the overexpression of laminins can be involved in the occurrence of inflammatory bowel disease-related cancers (Ljubimov et al., 2014).

Transcriptome results showed that the expression of the intestinal tight junction membrane protein CLDN4 is increased under the action of *C. butyricum* B14. QPCR results showed increased expression of mRNA of four other intestinal tight junction proteins, including claudin-3, occludin, ZO-1, and ZO-2. The barrier function of the intestinal epithelium is closely related to the expression of these functional proteins (Liévin-Le Moal and Servin, 2013), indicating that *C. butyricum* B14 treatment promoted the repair of the damaged intestinal barrier. Moreover, Ma et al. (2022) showed that *C. butyricum* and its derived extracellular vesicles upregulate tight junction-related protein genes. Tight junctions act as physical barriers that prevent solutes and water from freely passing through the paracellular space between the epithelial or endothelial cell sheets. They also play a crucial role in maintaining cell polarity and signal transduction (Ma et al., 2018). This suggests that the administration of *C. butyricum* B14 can enhance the function of the colonic barrier and reduce intestinal permeability more prominently than *C. butyricum* CB. These results were consistent with those of several studies that revealed the enhancement in the expression of intestinal barrier genes upon the addition of *C. butyricum* (Ma et al., 2012; Mao Hagihara et al., 2019; Zhao et al., 2020).

In addition, we observed a significant decrease in *Snai2* expression in the B14 and BC groups. SNAI2 (SNAIL2/Slug), encoded by *Snai2*, is an important regulator of epithelial-to-mesenchymal transition (EMT) and can directly act on e-cadherin (Serrano-Gomez et al., 2016). E-cadherin regulates cell-to-cell adhesion and maintains the structural and functional integrity of epithelial tissue (van Roy and Berx, 2008). *Snai2* also plays an important role in intestinal barrier protection. SNAI2 plays a crucial role in cancer progression (Emadi Baygi et al., 2010; Guo et al., 2022). Several studies have shown that *Snai2* expression is upregulated in enteritis (Zidar et al., 2016; Guo et al., 2023), which is consistent with our findings. When *C. butyricum* is added, the expression level of *Snai2* is significantly inhibited, whereas that of e-cadherin tends to be normal, intercellular adhesion is enhanced, the EMT process is blocked, and colon fibrosis is reduced (Guo et al., 2023). Fibrosis causes the colon to shorten and is an important sign of enteritis (Zidar et al., 2016). Therefore, our results show that the increase in colon length and the decrease in histological index after the addition of *C. butyricum* may reflect the decrease in fibrosis.

### 4.3 *Clostridium butyricum* can regulate the structure of the gut microbiota to decrease inflammation

The gut microbiota plays an important role in the host immune system, and an imbalance in the gut microbiota can disrupt the intestinal epithelial barrier and lead to the infiltration of inflammatory cytokines, thereby causing gut-related diseases (Neurath, 2019). Our results showed that *C. butyricum* significantly improved the gut microbiota, especially with strains B14 and CB (Figure 8).

At the genus level, after administration of *C. butyricum*, the relative abundance of *Alistips* decreased. *Alistipes* is considered a potential pathogen in the intestine, and may cause inflammation (Li et al., 2022). Similarly, Ma et al. (2022) showed that *C. butyricum* MIYAIRI II 588 could treat DSS-induced colitis by reducing levels of pathogenic bacteria in intestines. The relative abundance of *Akkermansia* increased in B14_14. *Akkermansia* stimulates mucus production and thickens the mucus layer to alleviate gut inflammation (Mithieux, 2018) and improves the intestinal barrier (Wang et al., 2020). This is consistent with the increased expression of *Muc4* shown in the transcriptome results. In addition, the main increase in CB_14 was the relative abundance of *Bifidobacterium* and *Faecalibaculum*.

After DSS administration, the relative abundance of the phylum Erysipelotrichaceae and genus *Dubosiella* was significantly increased in the DC group. Erysipelotrichaceae is harmful to intestinal diseases (He et al., 2021). Studies have shown that *Dubosiella* can act as a probiotic that could prevent enteritis (Zhai et al., 2019; Wan et al., 2022) and that *Dubosiella* can play a role in improving obesity and antiaging (Liu et al., 2023).

Both the B14 and CB groups showed increased abundances of *Bifidobacterium* and *Faecalibaculum*. *Bifidobacterium* is an important component of the human and animal gut microbiota (Biavati and Mattarelli, 2015) and has biological barrier and nutritional effects, antitumor properties, and can enhance the immune system, improve gut function, combat aging (Bahmani et al., 2019; Din et al., 2020; Pizzo et al., 2020; Yao et al., 2021), and treat depression (Tian et al., 2020, 2022). *Faecalibaculum* also plays a role in regulating gut microbiota by promoting metabolism, preventing obesity, and acting as an antitumor agent (Ivanov et al., 2009; Zagato et al., 2020). *Faecalibaculum* can produce short-chain fatty acids (e.g., butyric acid) that inhibit the production of inflammatory factors, promote the production of mucins as well as the synthesis of tight junction proteins and antimicrobial peptides, and strengthen the intestinal barrier (Hu, 2023; Li L. et al., 2023). The downregulation of *Tifa* and *Igkv12-89* and the upregulation of *Muc4*, *Lama3*, *Cldn4*, *Cldn3*, *Zo1, Zo2*, and *Snai2* may be caused by these factors.

## 5 Conclusion

Administration of *C. butyricum* isolated from giant panda feces inhibited inflammation of the small intestine in a mouse enteritis model and improved the morphology of the small intestine. This was achieved by increasing the expression of intestinal barrier proteins and thereby enhancing the integrity of the intestinal barrier to protect the intestine. Furthermore, by regulating the expression of key genes, the expression of proteins in related inflammatory pathways was inhibited and immune responses alleviated. Additionally, *C. butyricum* B14 treatment increased the abundance of certain probiotic species. Therefore, *C. butyricum* B14 could be used as an important probiotic for enteritis prevention and health protection.

## Data availability statement

The data presented in the study are deposited in the NCBI SRA repository; accession numbers SRX23459478 - SRX23459517 (16SrDNA) and SRX23374651- SRX23374662 (Transcriptome).

## Ethics statement

This study was reviewed and approved by the Institutional Animal Care and Use Committee (IACUC) of the Chengdu Research Base of Giant Panda Breeding (No. 2022004).

## Author contributions

SY: Data curation, Formal analysis, Investigation, Software, Validation, Writing – original draft, Writing – review & editing, Methodology, Resources, Visualization. JX: Writing – review & editing. QG: Writing – review & editing. XY: Writing – review & editing. YW: Writing – review & editing. TL: Writing – review & editing. LL: Writing – review & editing. JZ: Writing – review & editing, Funding acquisition, Supervision. WZ: Writing – review & editing, Funding acquisition, Methodology. XS: Writing – review & editing, Funding acquisition.

## Footnotes

^1^ https://May2021.archive.ensembl.org/Mus_musculus/Info/Index

^2^ https://www.microbiomeanalyst.ca

^3^ http://huttenhower.org/galaxy/
