# Supplementary material for: Clostridium butyricum isolated from giant panda can attenuate dextran sodium sulfate-induced colitis in mice
Source: Front Microbiol. 2024 Apr 5;15:1361945. doi: 10.3389/fmicb.2024.1361945 (PMC11027743; doi:10.3389/fmicb.2024.1361945)
Supplement: Supplementary file 1 [file Data_Sheet_1.PDF]

Supplementary Material

Clostridium butyricum derived from Giant Panda can attenuates DSS-induced Colitis of in mice following bettering intestinal mucosal barrier, modulating immune responses and improving gut microbiota

Shuran Yu, Jielong Zhou*, Junjin Xie, Qiang Guo, Yuxiang Wang, Tangjian Leng, Xia Yan, Lin Li, Wenping Zhang*, Xiaoyan Su*

*** Correspondence:** Corresponding Author: Jielong Zhou: zhoujielong1976@163.com, Wenping Zhang: zhang_zoology@163.com, Xiaoyan Su: xyansu@126.com

Table S1 Scoring system of disease activity index

Score	Weight loss(%)	Stool consistency	rectal bleeding
0	None	Normal	Normal
1	1-5	soft but firm	stool hemoccult positive
2	5-10	Soft	Hemoccult positive and visual pellet bleeding
3	>10	Diarrhea	gross bleeding around anus

DAI (Scoring system of disease activity index) score = (body weight loss score + fecal status score + rectal bleeding score) / 3

Table S2 Mouse colitis histology index scoring

Histological scores	Degree of inflammatory cell infiltration	Depth of inflammatory cell infiltration	Intestinal crypt destruction	Extent of lesions
0	None	None	None	0
1	A little bit	Mucosal layer	Basal 1/3 crypt destruction	1-25%
2	Serious	submucosa	Basal 2/3 crypt destruction	26-50%
3	Very serious	Muscular and serosal layers	Only the complete superficial epithelium	51-75%
4			Total crypt and epithelial destruction	76-100%

Table S3 RNA-seq read alignments

	Sample	Raw_reads	Clean_reads	Clean_bases	Error(%)	Q20(%)	Q30(%)	GC(%)
DC	B1	45370304	44284088	6.62G	0.04	98.23	93.66	48.27
DC	B2	45652694	44677366	6.68G	0.04	98.31	93.95	48.39
DC	B3	47190938	46181918	6.91G	0.04	98.28	93.85	48.31
DC	B4	50488272	49464870	7.39G	0.04	98.38	94.21	48.26
B14	D1	51648480	50567416	7.56G	0.04	98.36	94.12	48.53
B14	D2	48477570	47386146	7.08G	0.04	98.27	93.79	48.63
B14	D3	55350972	54260808	8.12G	0.04	98.36	94.08	48.59
B14	D4	45368240	44338878	6.62G	0.04	98.3	93.94	48.2
CB	E1	52486960	51358828	7.68G	0.04	98.27	93.77	48.51
CB	E2	47279018	46396468	6.94G	0.04	98.37	94.13	48.95
CB	E3	50070954	48748620	7.29G	0.04	98.12	93.3	48.11
CB	E4	58454404	57043838	8.53G	0.04	98.12	93.22	49.42

Table S4 *Claudin-3*, *Occludin*, *ZO-1*, *ZO-2* gene primer sequence

Gene	Gene primer sequence (5'-3')	NCBI Gene ID
Claudin-3	AACTGCGTACAAGACGAGACG	12739
	ATCCCTGATGATGGTGTTGG	
Occludin	CACACTTGCTTGGGACAGAG	18260
	TAGCCATAGCCTCCATAGCC	
ZO1	CTTCTCTTGCTGGCCCTAAAC	21872
	TGGCTTCACTTGAGGTTTCTG	
ZO2	ATGGGAGCAGTACACCGTGA	21873
	GACCACCCTGTCATTTTCTTG	
GAPDH	TCAAGAAGGTGGTGAAGCAG	14433
	AAGGTGGAAGAGTGGGAGTTG	

Table S5 Summary of de novo assembly of transcriptome sequence reads with reference genome

Sample	Raw reads	Clean_reads	Clean_bases	Error (%)	Q20 (%)	Q30 (%)	GC (%)	Total reads	Total mapped
B1	45370304	44284088	6.62G	0.04	98.23	93.66	48.27	44284088	43366753(97.93%)
B2	45652694	44677366	6.68G	0.04	98.31	93.95	48.39	44677366	43786460(98.01%)
B3	47190938	46181918	6.91G	0.04	98.28	93.85	48.31	46181918	45299436(98.09%)
D1	51648480	50567416	7.56G	0.04	98.36	94.12	48.53	50567416	49586137(98.06%)
D2	48477570	47386146	7.08G	0.04	98.27	93.79	48.63	47386146	46627151(98.40%)
D3	55350972	54260808	8.12G	0.04	98.36	94.08	48.59	54260808	53358248(98.34%)
E1	52486960	51358828	7.68G	0.04	98.27	93.77	48.51	51358828	50401795(98.14%)
E2	47279018	46396468	6.94G	0.04	98.37	94.13	48.95	46396468	45566908(98.21%)
E3	58454404	57043838	8.53G	0.04	98.12	93.22	49.42	57043838	55283683(96.91%)

Table S6 The groups of 16S and 16S rRNA gene sequences

groups	sample-id	input	filtered	percentage of input passed filter	denoised	merged	percentage of input merged	non-chimeric	percentage of input non-chimeric	Total_ASVs	ASV_counts
BC_14	A1_14	136625	134392	98.37	129783	97622	71.45	49910	36.53	7975	754
BC_14	A2_14	145285	143307	98.64	138448	106583	73.36	55182	37.98	7975	775
BC_14	A3_14	143402	141225	98.48	138012	120070	83.73	65677	45.8	7975	582
BC_14	A4_14	145903	143739	98.52	140675	122262	83.8	77244	52.94	7975	644
DC_14	B1_14	136333	134052	98.33	131360	117765	86.38	59929	43.96	7975	458
DC_14	B2_14	139963	138050	98.63	135418	122766	87.71	72151	51.55	7975	476
DC_14	B3_14	146372	144280	98.57	141373	126847	86.66	85294	58.27	7975	544
DC_14	B4_14	145129	142957	98.5	140568	127831	88.08	68623	47.28	7975	409
B13_14	C1_14	137493	135522	98.57	133710	125337	91.16	102215	74.34	7975	445
B13_14	C2_14	136652	134566	98.47	130644	106486	77.92	53396	39.07	7975	663
B13_14	C3_14	147050	144795	98.47	141540	123631	84.07	71363	48.53	7975	598
B13_14	C4_14	141372	139301	98.54	134749	102160	72.26	51442	36.39	7975	802
B14_14	D1_14	146557	144187	98.38	140824	123875	84.52	71577	48.84	7975	543
B14_14	D2_14	148006	145525	98.32	141463	120642	81.51	60508	40.88	7975	626
B14_14	D3_14	142089	139960	98.5	135899	111552	78.51	63809	44.91	7975	759
B14_14	D4_14	137402	135095	98.32	132768	118934	86.56	64930	47.26	7975	454
CB_14	E1_14	147587	145216	98.39	140525	112654	76.33	62470	42.33	7975	809
CB_14	E2_14	140574	138113	98.25	135342	122641	87.24	61373	43.66	7975	420
CB_14	E3_14	136890	134758	98.44	132269	118873	86.84	54781	40.02	7975	474
CB_14	E4_14	139870	137637	98.4	135578	125094	89.44	64881	46.39	7975	377

BC_21	A1_21	145933	143266	98.17	138785	110517	75.73	49982	34.25	7975	774
BC_21	A2_21	135174	133373	98.67	128607	97886	72.41	46909	34.7	7975	750
BC_21	A3_21	149018	146764	98.49	141724	108513	72.82	51350	34.46	7975	816
BC_21	A4_21	143128	140744	98.33	138455	123930	86.59	65554	45.8	7975	378
DC_21	B1_21	135324	133126	98.38	131519	122489	90.52	78169	57.76	7975	245
DC_21	B2_21	148229	146086	98.55	144936	137762	92.94	97558	65.82	7975	251
DC_21	B3_21	138158	135781	98.28	133938	124727	90.28	73829	53.44	7975	247
DC_21	B4_21	137313	135763	98.87	134358	128894	93.87	87899	64.01	7975	228
B13_21	C1_21	143130	141467	98.84	140423	136730	95.53	105760	73.89	7975	217
B13_21	C2_21	135561	133636	98.58	131880	122741	90.54	83652	61.71	7975	293
B13_21	C3_21	136106	134471	98.8	133527	130346	95.77	96237	70.71	7975	181
B13_21	C4_21	145730	143641	98.57	141605	132558	90.96	95891	65.8	7975	311
B14_21	D1_21	140429	138769	98.82	137669	131923	93.94	82784	58.95	7975	195
B14_21	D2_21	137716	135854	98.65	134553	128420	93.25	82678	60.04	7975	203
B14_21	D3_21	137663	135215	98.22	133495	126645	92	82776	60.13	7975	296
B14_21	D4_21	136210	134254	98.56	132596	125612	92.22	77073	56.58	7975	261
CB_21	E1_21	147270	144992	98.45	143186	133580	90.7	78938	53.6	7975	323
CB_21	E2_21	143776	140072	97.42	138636	130878	91.03	82930	57.68	7975	258
CB_21	E3_21	140101	137333	98.02	135905	128808	91.94	75716	54.04	7975	301
CB_21	E4_21	144766	143035	98.8	142171	139137	96.11	93904	64.87	7975	184

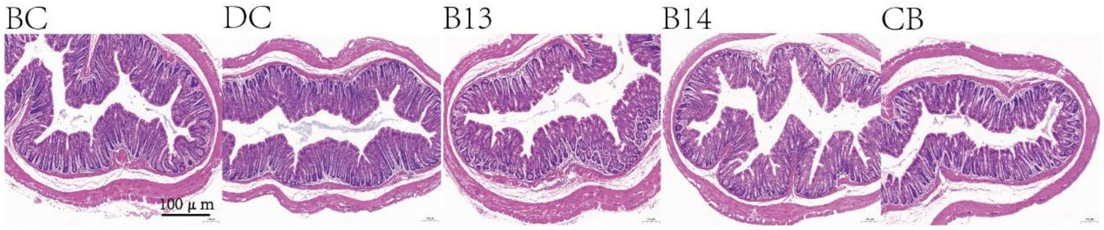

Supplementary Figure 1. Hematoxylin and eosin staining of colonic tissues on 14th day.

(A)

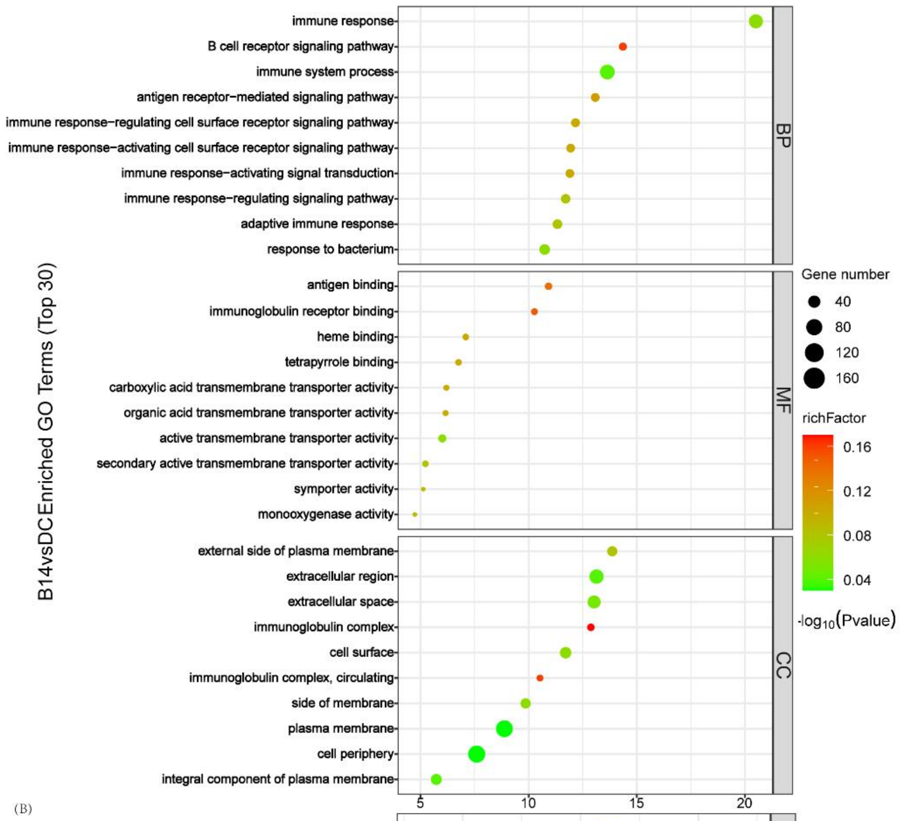
B14vsDCEnriched GO Terms (Top 30)
immune response
B cell receptor signaling pathway
immune system process
antigen receptor−mediated signaling pathway
immune response−regulating cell surface receptor signaling pathway
immune response−activating cell surface receptor signaling pathway
immune response−activating signal transduction
immune response−regulating signaling pathway
adaptive immune response
response to bacterium
antigen binding
immunoglobulin receptor binding
heme binding
tetrapyrrole binding
carboxylic acid transmembrane transporter activity
organic acid transmembrane transporter activity
active transmembrane transporter activity
secondary active transmembrane transporter activity
symporter activity
monooxygenase activity
external side of plasma membrane
extracellular region
extracellular space
immunoglobulin complex
cell surface
immunoglobulin complex, circulating
side of membrane
plasma membrane
cell periphery
integral component of plasma membrane
BP
MF
CC
5
10
15
20
-log10(Pvalue)
Gene number
40
80
120
160
richFactor
0.16
0.12
0.08
0.04

(B)

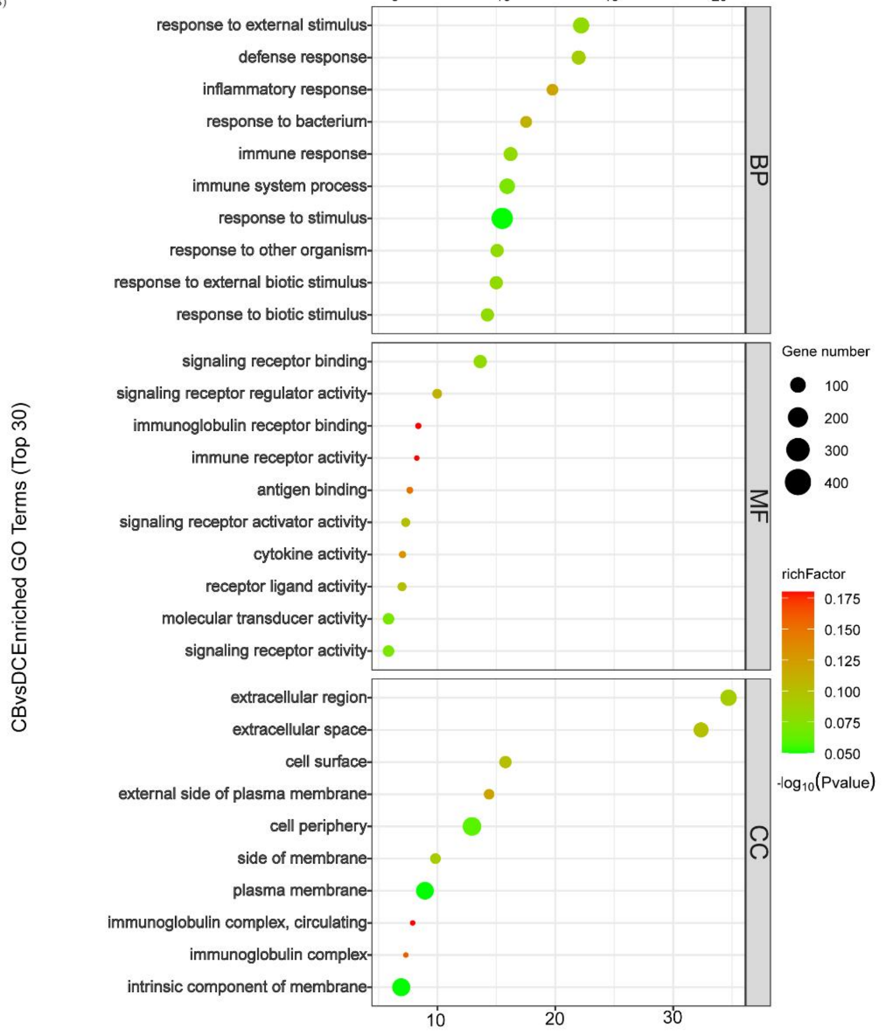
CBvsDCEnriched GO Terms (Top 30)
response to external stimulus
defense response
inflammatory response
response to bacterium
immune response
immune system process
response to stimulus
response to other organism
response to external biotic stimulus
response to biotic stimulus
signaling receptor binding
signaling receptor regulator activity
immunoglobulin receptor binding
immune receptor activity
antigen binding
signaling receptor activator activity
cytokine activity
receptor ligand activity
molecular transducer activity
signaling receptor activity
extracellular region
extracellular space
cell surface
external side of plasma membrane
cell periphery
side of membrane
plasma membrane
immunoglobulin complex, circulating
immunoglobulin complex
intrinsic component of membrane
BP
MF
CC
10
20
30
-log10(Pvalue)
Gene number
100
200
300
400
richFactor
0.175
0.150
0.125
0.100
0.075
0.050

Supplementary Figure 2. The top 30 enrichment analysis of GO term based on the DEGs in B14 vs. DC (A) and CB vs. DC (B).

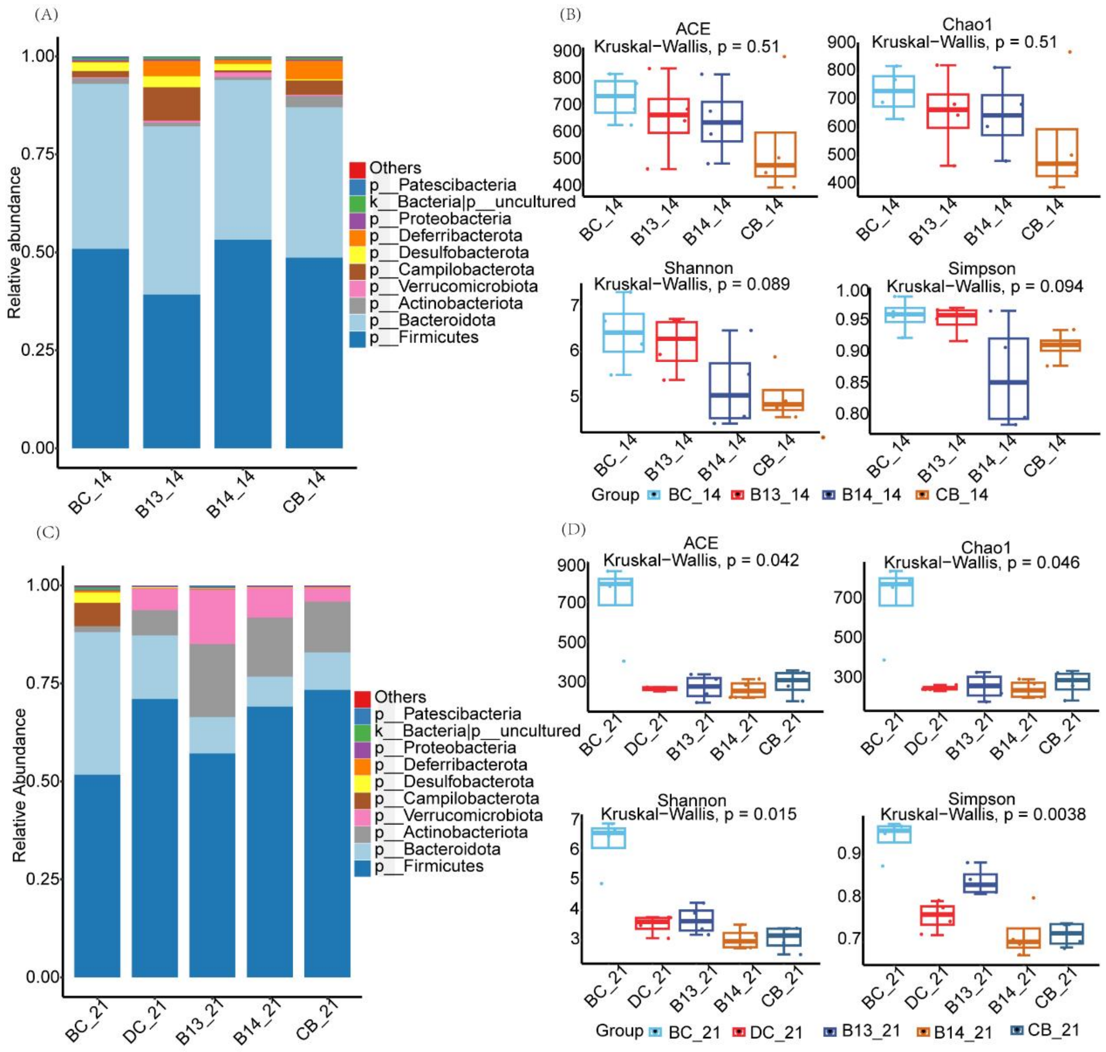

Supplementary Figure 3. C. butyricum supplementation modulated the composition of colonic microbiota. At the phylum level, the heatmap of the abundant colonic microbiota on the 14th day (A) and five groups on the 21th day (C). The α-diversity of four groups on the 14th day (B) and five groups on the 21th day (D).

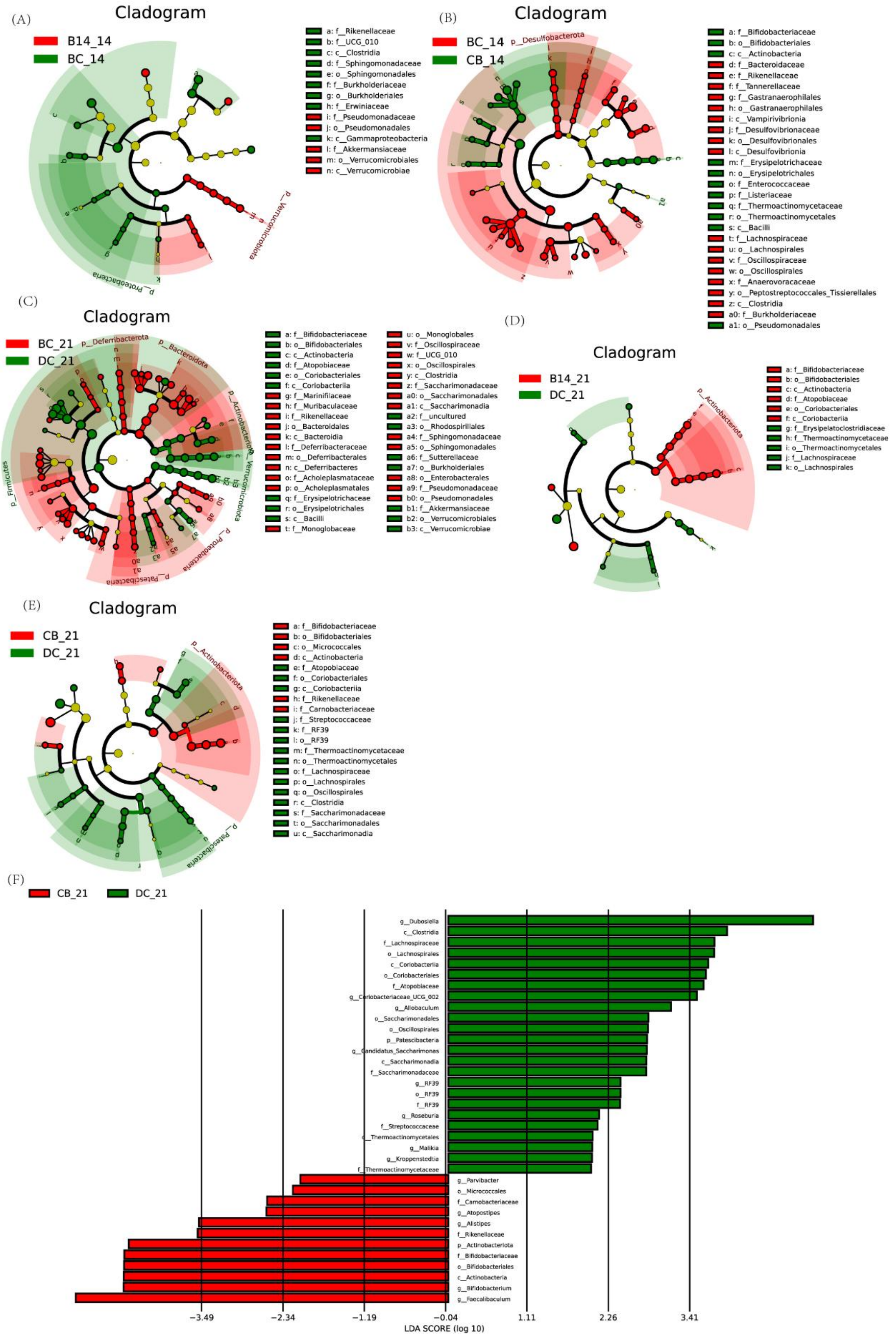
(A)
Cladogram
B14_14
BC_14
a: f__Rikenellaceae
b: f__UCG_010
c: c__Clostridia
d: f__Sphingomonadaceae
e: o__Sphingomonadales
f: f__Burkholderiaceae
g: o__Burkholderiales
h: f__Erwiniaceae
i: f__Pseudomonadaceae
j: o__Pseudomonadales
k: c__Gammaproteobacteria
l: f__Akkermansiaceae
m: o__Verrucomicrobiales
n: c__Verrucomicrobiae
p__Verrucomicrobiota
p__Proteobacteria
(B)
Cladogram
BC_14
CB_14
p__Desulfobacterota
a: f__Bifidobacteriaceae
b: o__Bifidobacteriales
c: c__Actinobacteria
d: f__Bacteroidaceae
e: f__Rikenellaceae
f: f__Tannerellaceae
g: f__Gastranaerophilales
h: o__Gastranaerophilales
i: c__Vampirivibrionia
j: f__Desulfovibrionaceae
k: o__Desulfovibrionales
l: c__Desulfovibrionia
m: f__Erysipelotrichaceae
n: o__Erysipelotrichales
o: f__Enterococcaceae
p: f__Listeriaceae
q: f__Thermoactinomycetaceae
r: o__Thermoactinomycetales
s: c__Bacilli
t: f__Lachnospiraceae
u: o__Lachnospirales
v: f__Oscillospiraceae
w: o__Oscillospirales
x: f__Anaerovoracaceae
y: o__Peptostreptococcales_Tissierellales
z: c__Clostridia
a0: f__Burkholderiaceae
a1: o__Pseudomonadales
(C)
Cladogram
BC_21
DC_21
p__Deferribacterota
p__Bacteroidota
p__Actinobacteriota
p__Verrucomicrobiota
p__Firmicutes
p__Proteobacteria
p__Patescibacteria
a: f__Bifidobacteriaceae
b: o__Bifidobacteriales
c: c__Actinobacteria
d: f__Atopobiaceae
e: o__Coriobacteriales
f: c__Coriobacteriia
g: f__Marinifilaceae
h: f__Muribaculaceae
i: f__Rikenellaceae
j: o__Bacteroidales
k: c__Bacteroidia
l: f__Deferribacteraceae
m: o__Deferribacterales
n: c__Deferribacteres
o: f__Acholeplasmataceae
p: o__Acholeplasmatales
q: f__Erysipelotrichaceae
r: o__Erysipelotrichales
s: c__Bacilli
t: f__Monoglobaceae
u: o__Monoglobales
v: f__Oscillospiraceae
w: f__UCG_010
x: o__Oscillospirales
y: c__Clostridia
z: f__Saccharimonadaceae
a0: o__Saccharimonadales
a1: c__Saccharimonadia
a2: f__uncultured
a3: o__Rhodospirillales
a4: f__Sphingomonadaceae
a5: o__Sphingomonadales
a6: f__Sutterellaceae
a7: o__Burkholderiales
a8: o__Enterobacterales
a9: f__Pseudomonadaceae
b0: o__Pseudomonadales
b1: f__Akkermansiaceae
b2: o__Verrucomicrobiales
b3: c__Verrucomicrobiae
(D)
Cladogram
B14_21
DC_21
p__Actinobacteriota
a: f__Bifidobacteriaceae
b: o__Bifidobacteriales
c: c__Actinobacteria
d: f__Atopobiaceae
e: o__Coriobacteriales
f: c__Coriobacteriia
g: f__Erysipelatoclostridiaceae
h: f__Thermoactinomycetaceae
i: o__Thermoactinomycetales
j: f__Lachnospiraceae
k: o__Lachnospirales
(E)
Cladogram
CB_21
DC_21
p__Actinobacteriota
p__Patescibacteria
a: f__Bifidobacteriaceae
b: o__Bifidobacteriales
c: o__Micrococcales
d: c__Actinobacteria
e: f__Atopobiaceae
f: o__Coriobacteriales
g: c__Coriobacteriia
h: f__Rikenellaceae
i: f__Carnobacteriaceae
j: f__Streptococcaceae
k: f__RF39
l: o__RF39
m: f__Thermoactinomycetaceae
n: o__Thermoactinomycetales
o: f__Lachnospiraceae
p: o__Lachnospirales
q: o__Oscillospirales
r: c__Clostridia
s: f__Saccharimonadaceae
t: o__Saccharimonadales
u: c__Saccharimonadia
(F)
CB_21
DC_21
g__Dubosiella
c__Clostridia
f__Lachnospiraceae
o__Lachnospirales
c__Coriobacteriia
o__Coriobacteriales
f__Atopobiaceae
g__Coriobacteriaceae_UCG_002
g__Allobaculum
o__Saccharimonadales
o__Oscillospirales
p__Patescibacteria
g__Candidatus_Saccharimonas
c__Saccharimonadia
f__Saccharimonadaceae
g__RF39
o__RF39
f__RF39
g__Roseburia
f__Streptococcaceae
o__Thermoactinomycetales
g__Mailkia
g__Kroppenstedtia
f__Thermoactinomycetaceae
g__Parvibacter
o__Micrococcales
f__Carnobacteriaceae
g__Atopostipes
g__Alistipes
f__Rikenellaceae
p__Actinobacteriota
f__Bifidobacteriaceae
o__Bifidobacteriales
c__Actinobacteria
g__Bifidobacterium
g__Faecalibaculum
−3.49
−2.34
−1.19
−0.04
1.11
2.26
3.41
LDA SCORE (log 10)

(G)

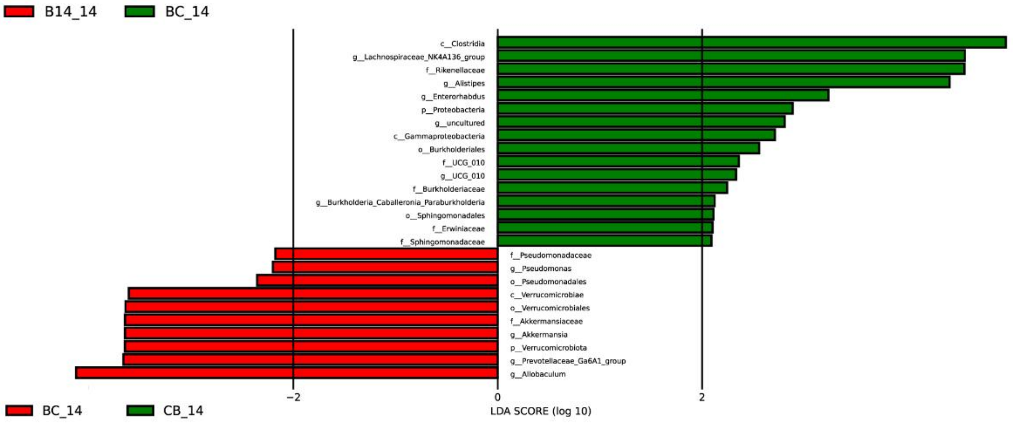
B14_14
BC_14
c__Clostridia
g__Lachnospiraceae_NK4A136_group
f__Rikenellaceae
g__Alistipes
g__Enterorhabdus
p__Proteobacteria
g__uncultured
c__Gammaproteobacteria
o__Burkholderiales
f__UCG_010
g__UCG_010
f__Burkholderiaceae
g__Burkholderia_Caballeronia_Paraburkholderia
o__Sphingomonadales
f__Erwiniaceae
f__Sphingomonadaceae
f__Pseudomonadaceae
g__Pseudomonas
o__Pseudomonadales
c__Verrucomicrobiae
o__Verrucomicrobiales
f__Akkermansiaceae
g__Akkermansia
p__Verrucomicrobiota
g__Prevotellaceae_Ga6A1_group
g__Allobaculum
−2
0
2
LDA SCORE (log 10)

(H)

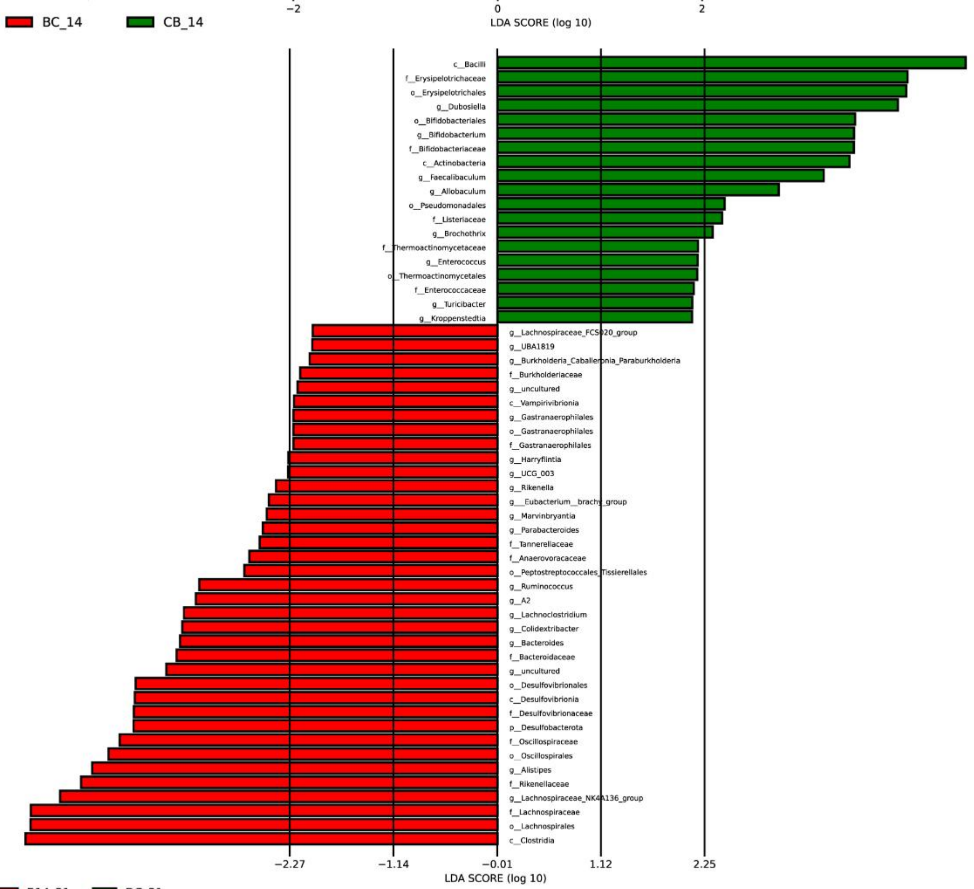
BC_14
CB_14
c__Bacilli
f__Erysipelotrichaceae
o__Erysipelotrichales
g__Dubosiella
o__Bifidobacteriales
g__Bifidobacterium
f__Bifidobacteriaceae
c__Actinobacteria
g__Faecalibaculum
g__Allobaculum
o__Pseudomonadales
f__Listeriaceae
g__Brochothrix
f__Thermoactinomycetaceae
g__Enterococcus
o__Thermoactinomycetales
f__Enterococcaceae
g__Turicibacter
g__Kroppenstedtia
g__Lachnospiraceae_FCS020_group
g__UBA1819
g__Burkholderia_Caballeronia_Paraburkholderia
f__Burkholderiaceae
g__uncultured
c__Vampirivibrionia
g__Gastranaerophilales
o__Gastranaerophilales
f__Gastranaerophilales
g__Harryflintia
g__UCG_003
g__Rikenella
g___Eubacterium__brachy_group
g__Marvinbryantia
g__Parabacteroides
f__Tannerellaceae
f__Anaerovoracaceae
o__Peptostreptococcales_Tissierellales
g__Ruminococcus
g__A2
g__Lachnoclostridium
g__Colidextribacter
g__Bacteroides
f__Bacteroidaceae
g__uncultured
o__Desulfovibrionales
c__Desulfovibrionia
f__Desulfovibrionaceae
p__Desulfobacterota
f__Oscillospiraceae
o__Oscillospirales
g__Alistipes
f__Rikenellaceae
g__Lachnospiraceae_NK4A136_group
f__Lachnospiraceae
o__Lachnospirales
c__Clostridia
−2.27
−1.14
−0.01
1.12
2.25
LDA SCORE (log 10)

(I)

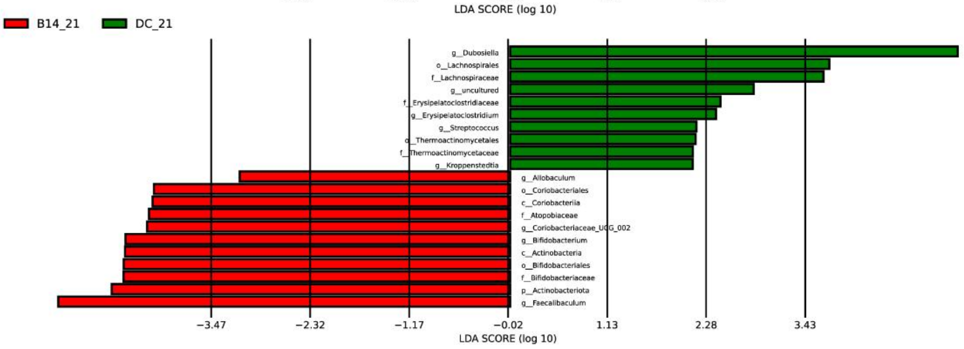
B14_21
DC_21
g__Dubosiella
o__Lachnospirales
f__Lachnospiraceae
g__uncultured
f__Erysipelatoclostridiaceae
g__Erysipelatoclostridium
g__Streptococcus
o__Thermoactinomycetales
f__Thermoactinomycetaceae
g__Kroppenstedtia
g__Allobaculum
o__Coriobacteriales
c__Coriobacteriia
f__Atopobiaceae
g__Coriobacteriaceae_UCG_002
g__Bifidobacterium
c__Actinobacteria
o__Bifidobacteriales
f__Bifidobacteriaceae
p__Actinobacteriota
g__Faecalibaculum
−3.47
−2.32
−1.17
−0.02
1.13
2.28
3.43
LDA SCORE (log 10)

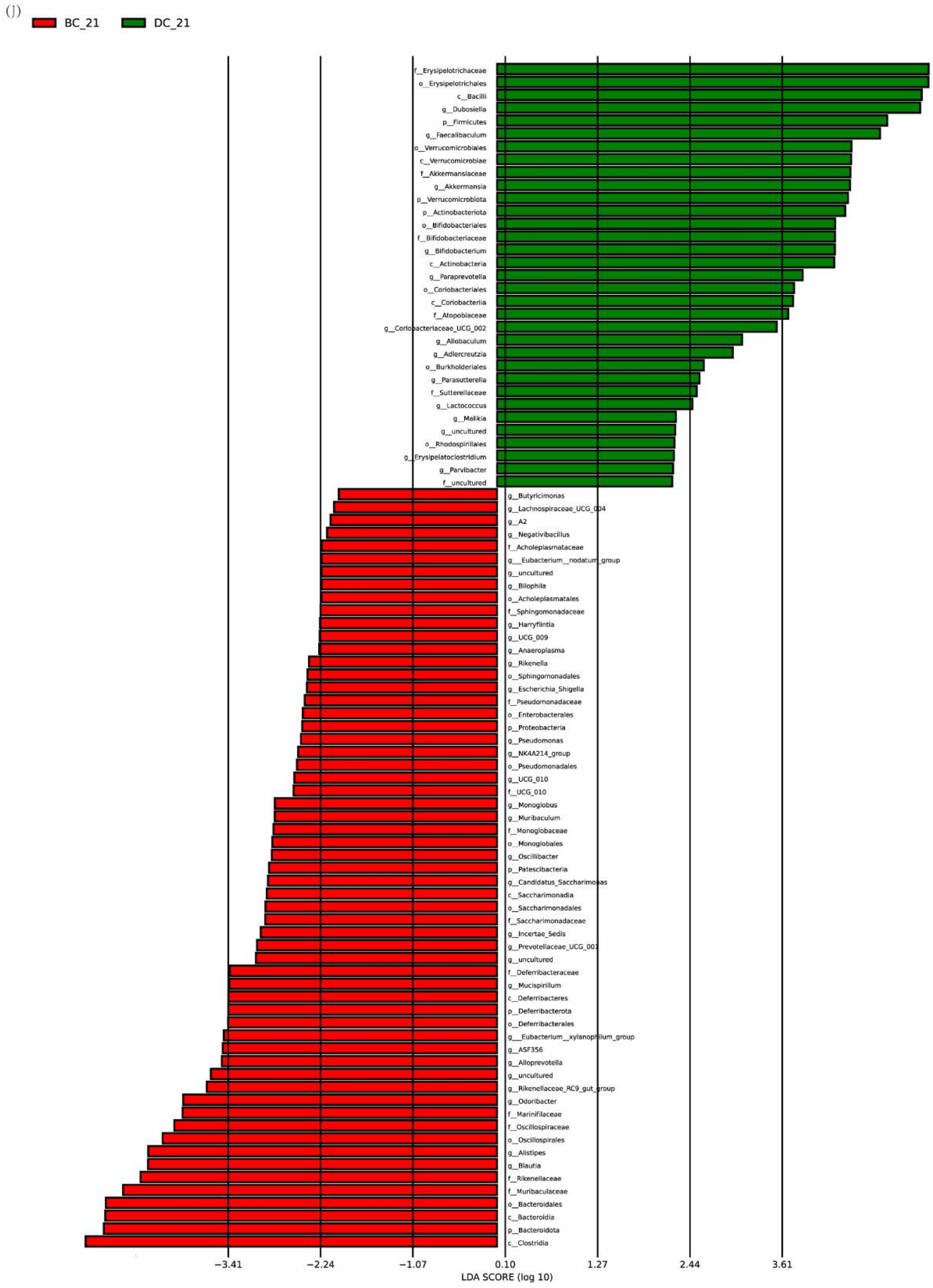

Supplementary Figure 4. The results of the LefSe analysis include the evolutionary branch plot in BC_14 vs. B14_14 (A), BC_14 vs. CB_14 (B), BC_21 vs. DC_21 (C), B14_21 vs. DC_21 (D), CB_21 vs. DC_21 (E) and the LDA value distribution histogram in BC_14 vs. B14_14 (G), BC_14 vs. CB_14 (H), BC_21 vs. DC_21 (J), B14_21 vs. DC_21 (I), CB_ 21vs. DC_21 (F). (LDA > 2)
